# Sphingosine-1-Phosphate Receptor 4 Attenuates Neutrophilic Airway Inflammation in Experimental Asthma via Repressing Proinflammatory Macrophage Activation

**DOI:** 10.7150/ijbs.80256

**Published:** 2023-03-05

**Authors:** Shanshan Wang, Zhen Tian, Yanjiao Lu, Zhenli Huang, Yan Fan, Boyu Li, Hongyan Zheng, Xiaojie Wu, Meijia Wang, Jianping Zhao, Jungang Xie

**Affiliations:** 1 Department of Respiratory and Critical Care Medicine, National Clinical Research Center of Respiratory Disease, Key Laboratory of Pulmonary Diseases of Health Ministry, Tongji Hospital, Tongji Medical College, Huazhong University of Science and Technology, Wuhan, Hubei, 430030, China; 2 Department of Respiratory and Critical Care Medicine, Wuhan NO.1 Hospital, Wuhan Hospital of traditional Chinese and Western Medicine, Wuhan 430022, China

**Keywords:** sphingosine metabolism, S1PR4, macrophage, FPR2, neutrophilic asthma

## Abstract

Patients with eosinophilic asthma react well to conventional treatment of asthma while individualized therapy for non-eosinophilic endotypes have yet to be developed. Dysregulated sphingosine metabolites are associated with the pathophysiology of different asthma endotypes with their receptors involved. However, whether the sphingosine-1-phosphate receptor 4 (S1PR4) contributes to disease progression of asthma remains underappreciated. In this study, we demonstrated that sphingosine metabolism was disturbed in asthma while it could not be used to distinguish between different endotypes of asthma. S1PR4, a vital receptor of bioactive sphingosine metabolites and mainly expressed in macrophages, exhibited lower expression both in patients and experimental mice with neutrophilic airway inflammation. Additionally, *S1pr4* deficiency aggravated the OVA/LPS-induced pulmonary inflammation in mice along with a significant up-regulation in M1 macrophage activation. Mechanistic studies showed that S1PR4 was strongly connected to bioactive oxylipins concurrent with bounding to formyl peptide receptor 2 to influence the phosphorylation of JNK and contributed to the macrophage M1 program, which in turn secreted amounts of inflammatory cytokines associated to the inflammatory response of neutrophilic asthma. Furthermore, treating mice with S1PR4 agonist CYM50308 was characterized by less pulmonary inflammatory infiltration. Our research indicates S1PR4 a promising therapeutic target for non-eosinophilic phenotypes of asthma.

## Introduction

Asthma is a heterogeneous disease characterized by chronic inflammation in the airway and affects over 350 million people worldwide. Although a decline in hospitalization and deaths from asthma has arisen in some countries, a significant subpopulation of patients is refractory to conventional treatments, putting a heavy burden on families of patients and health care systems 1-4. Asthma can be classified into different inflammatory endotypes according to the sputum analyses 5. Despite the classical perspective that eosinophilic inflammation and type 2 response play a dominant role in asthma pathogenesis, it is present in only half of asthmatic patients 6. On the other hand, non-eosinophilic airway inflammation including neutrophilic asthma (NA) manifests as a persistent, more severe, and corticosteroid-resistant disease state 7.

Sphingolipids, building blocks of eukaryotic cell membranes, are a large group of lipids ubiquitously distributed in both animals and plants 8. Research has shown that childhood asthma with an onset early in life is connected to a disturbance of sphingolipid metabolism and other studies have also indicated that sphingolipids and their metabolites could be related to the pathophysiology of asthma 9-11. Among sphingolipid, sphingosine metabolites have a relatively simple structure and are widely studied. Sphingosine and its bioactive mediator sphingosine-1-phosphate (S1P) play a role in numerous essential biological processes including cell growth, differentiation, senescence, and programmed cell death, which are significant in inflammation, immunity, and inflammatory disorders such as asthma 9, 12.

S1P and some other bioactive sphingosine metabolites (such as dihydro-S1P (dhS1P) and phytosphingosine-1-phosphate (P1P)) are predominantly mediated in a receptor dependent way, through G-protein coupled receptors (GPCRs) including S1PR1-5, following activating of several downstream signaling pathways 13, 14. S1PR1-3 are essentially widely distributed, whereas S1PR4 and S1PR5 are mainly exclusively expressed in specific cell types 15. Among the S1PRs confirmed of binding to different G protein's subunits 16, S1PR1-3 are well-characterized in the processes of various diseases, whereas the function of S1PR4 has been underappreciated 17-19. In consideration of the observation that S1PR4 is particularly abundant in immune cells 20, its possible role in inflammatory diseases is well worth exploring.

## Material and Methods

### Human subjects

For the untargeted lipidomics analysis, patients with asthma (n = 24) and healthy control (HC) subjects (n = 20) were enrolled from Tongji Hospital (Wuhan, China) as previously reported 21. Blood specimen and induced sputum from eosinophilic asthma (EA) patients (n = 16), non-eosinophilic asthma (NEA) patients (n = 15), and HC subjects (n = 10) were collected at Tongji Hospital. Plasma, peripheral blood mononuclear cells (PBMC), supernatant and smear of induced sputum were collected. Asthma diagnosis was confirmed based on the 2022 Global Initiative for Asthma (GINA) guidelines 1. Patients were divided into different endotypes according to a cut-off point of 3% eosinophils in the induced sputum. The present study was performed in accordance with the Declaration of Helsinki and approved by the Human Assurance Committee of Tongji Hospital. The contents of sphingosine metabolites and oxylipins as mentioned later in the article were identified by MetWare (http://www.metware.cn/) based on the AB Sciex QTRAP 6500 LC-MS/MS platform. Clinical data of targeted lipidomics analysis is provided in **Table S1**.

### Murine model

*S1pr4* knockout (*S1pr4*-KO) mice (JAX stock #005799) were procured from Jackson laboratory (Bar Harbor, Maine, United States). Wild type (WT) (C57BL/6) mice were procured from the Animal Experimental Center of Hubei province (Wuhan, China). The mice were all maintained in a specific pathogen-free animal center at the Tongji Hospital and subjected to a 12 h light-cycle environment with clean water and irradiated food. All the experiments were approved by the Animal Care and Use Committee of Tongji Hospital.

Mice were sensitized intranasally (i.n.) with ovalbumin (OVA) and lipopolysaccharide (LPS) in saline on days 0, 1, 2, and 13. Mice were infected with adenovirus (Obio Technology, Shanghai, China) expressing *S1pr4* (*S1pr4*-OE) or control vector on day 18 before the challenge as described hereafter when research needed. From days 20 to 22, all mice were challenged to daily intranasal administration of OVA or saline, as reported previously with a few alterations 22, 23. In addition, experimental mice were intraperitoneally (i.p.) administered with S1PR4 agonist CYM50308 or the same volume of cosolvent SBE-β-CD (Vehicle) the day before challenge and each time prior to the challenge starting on day 19 when necessary. For eosinophilic airway inflammation, mice were sensitized with OVA and Aluminum hydroxide (Alum) on days 0, 7 and 13. All mice were challenged the same as OVA/LPS induction, in a higher dose. All mice were sacrificed 24 h following last challenge for analysis.

### Reagents and antibodies

Sphingosine, sphinganine, S1P, dhS1P, P1P, OVA grade V and LPS were procured from Sigma-Aldrich (St. Louis, MO, USA). Murine recombinant IFN-γ was obtained from PeproTech (London, UK). Antibody against S1PR4 and mouse IL-1β, IL-6, and IL-12b ELISA kit were procured from Boster (Wuhan, Hubei, China). Fluorescent antibody against S1PR4 was obtained from Affinity Biosciences (Cincinnati, OH, USA). Antibodies against iNOS and formyl peptide receptor 2 (FPR2) were purchased from Abcam (Cambridge, MA, USA). Antibodies against p38, p-p38, p65, p-p65, JNK, P-JNK, Erk, p-Erk, FLAG and magnetic beads were procured from Cell Signaling Technology (Danvers, MA, USA). Antibodies against CD68 was purchased from Santa Cruz Biotechnology (Santa Cruz, CA, USA). Antibodies against Arg-1, IL-1β, and β‐Actin were procured from the Proteintech Group (Wuhan, Hubei, China). Anti-mouse CD45-APC/Cy7, F4/80-PE, CD11b-V450, and CD86-FITC were procured from BD Biosciences (San Jose, CA, USA). Anti-mouse CD206-PE/Cy7 and murine recombinant IL-4 were procured from BioLegend (San Diego, CA, USA). CYM50358 was obtained from Tocris Bioscience (Bristol, UK). All other reagents were obtained from MedChem Express (Monmouth Junction, NJ, USA), except stated otherwise.

### Histological and immunostaining analysis

The left lung was first inflated and transferred for 24 h to fresh 4% neutral-buffered paraformaldehyde at standard room temperature, then we performed paraffin embedding and histological analysis as reported previously. Anti-S1PR4 was used for immunostaining of lung tissue sections as described in the manufacturer's instructions. For immunofluorescence, the lung sections and smear were probed with the first antibody and then the fluorescent secondary antibodies of corresponding species were used for staining (Servicebio, Wuhan, China). Nuclei were counterstained blue with 4'-6-diamidino-2-phenylindole (DAPI). Images were scanned with fluorescence (Olympus, Melville, NY, USA) as well as a confocal microscope (Nikon, Tokyo, Japan).

### ELISA

The concentration of IL-6, IL-1β, and IL-12b in the bronchoalveolar lavage fluid (BALF) and cell culture supernatant was determined with the ELISA kits described above following the manufacturer's instructions.

### Culture and treatment of primary macrophages

Primary bone marrow derived macrophages (BMDMs) were procured from male mice as previously reported 24. The red blood cells contained in samples of bone marrow were first lysed and then resuspended in culture medium supplemented by 10% fetal bovine serum, RPMI 1640, penicillin and streptomycin, as well as macrophage colony-stimulating factor (M-CSF). Subsequently, the cells were plated at 37°C in 35×15 mm tissue culture dishes, while the medium was renewed every two days. Seven days thereafter, the differentiated macrophages, which were either infected with adenovirus or not, were cocultured with LPS/IFN-γ and CYM50308/CYM50358 of certain concentration if needed at the indicated time points.

Primary lung macrophages were obtained from both *S1pr4*-KO and WT mice. When transferred into single cell suspension of mice lung tissue by sterile collagenase I, cells were placed in 35×15 mm tissue culture dishes leaving to set for about 1-2 h and then the medium was changed. LPS/IFN-γ or IL-4 were administrated the following day.

### Western blot analysis

RIPA lysis buffer was applied to homogenize the lung tissues and cultured cells. Then the proteins underwent western blotting with the primary antibodies mentioned above by established techniques. To analyze the gray values, Image J software was used.

### Quantitative RT-PCR analysis

TRlzol reagent was applied for the isolation of total RNA from lung tissues and macrophages following the manufacturers' instructions. A reverse transcriptase kit was used to conduct complementary DNA synthesis. The SYBR Premix Ex Taq was used for quantitative RT-PCR and β-actin was used to normalize the relative expression of every target gene as reported previously. All the reagents mentioned above were obtained from Takara (Tokyo, Japan). **Table S2** shows the primers that were used for each target gene.

### Flow cytometry

Previously established techniques were used to isolate single cells from mice lung tissue 25. The dead cells were excluded with Zombie Aqua (BioLegend; San Diego, CA, USA), an amine-reactive fluorescent viability dye, according to the recommendations of the companies. Then the cells were stained with CD45-APC/Cy7 (1:100), F4/80-PE (1:100), CD11b-V450 (1:100), CD86-FITC (1:100), and CD206-PE/Cy7 (1:100) antibodies. Following washing, a cytoFlex (Beckman, Brea, CA, USA) was used to analyze the cells. FlowJo V10 software was applied to evaluate all the obtained data following the manufacturer's instructions.

### Comprehensive bioinformatics analysis

To identify *S1PR4*-related asthma phenotype genes, we used microarray analysis datasets from the Gene Expression Omnibus (GEO) database as described following. The heatmap analyses were performed with R software (version 4.1.3), and correlation clustering heatmap was analyzed with Metaboanalyst 3.0 (Montr´eal, QC, Canada). Network diagram of interrelated differentially expressed genes (DEGs) were performed by Cytoscape (version 3.9.1). Metabolic pathway analysis of oxylipins and their critical lipid metabolizing enzymes were based on the Kyoto Encyclopedia of Genes and Genomes (KEGG) database.

### Coimmunoprecipitation assay

Whole-cell lysate was used to perform immunoprecipitation as described in a previous protocol 26. Briefly, anti-FLAG antibody was used for the forming of immune complexes with the FPR2 protein in lysates at 4°C overnight. Then the complexes were immunoprecipitated for 2 h at 4°C with magnetic beads. Lysis buffer was used to wash the beads three times and SDS sample buffer was used to elute the bound proteins. Lastly, equivalent samples underwent western blot analysis for FLAG and FPR2.

### Statistical analysis

Data as a result of the experiments are reported as mean ± standard error of the mean (SEM). GraphPad Prism v7.0 software was used for comparisons between groups of at least three independent replications. Comparisons between two experimental groups were performed with Student's t test in case of paired data or Student's t test with Welch's correction in case of unpaired data. For comparisons between three groups or more, one-way ANOVA with Bonferroni's correction was applied. In all analyses, p< 0.05 was defined as statistical significance and expressed as ^*^ p<0.05; ^**^ p<0.01; ^***^ p<0.001; and ^****^ p<0.0001.

## Results

### Disturbance of sphingosine metabolism in asthma patients

**Figure 1:**
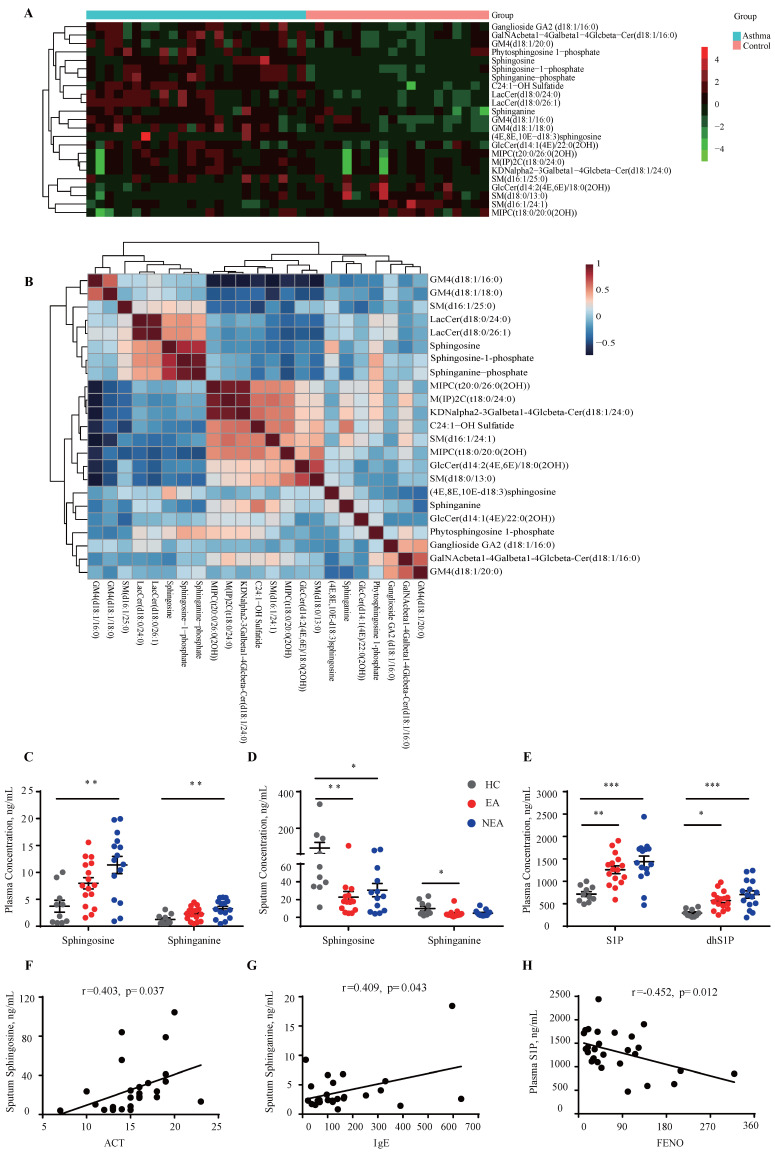
** Identification of the concentration variation of sphingosine metabolites in asthma patients. A:** Heatmap analysis of sphingolipids using untargeted lipidomics analysis. The colors in the heatmap depict the fold enrichment in each sample. **B:** Correlation clustering‐heatmap of sphingolipids using untargeted lipidomics analysis. The colors in the heatmap depict the fold enrichment in each sample. **C-D:** Targeted lipidomics analysis of sphingosine and sphinganine concentration in the plasma (C) and induced sputum (D) of asthma patients with different endotypes. **E:** Targeted lipidomics analysis of bioactive phosphorylated sphingosine metabolites concentration in the plasma of asthma patients with different endotypes. HC=healthy controls (n=10), EA=eosinophilic asthma patients (n=16), NEA=non-eosinophilic asthma patients (n=15). **F-H:** Correlation analysis between sphingosine metabolites and clinical characteristics of asthma patients. ACT=Asthma Control Test, IgE=serum total IgE concentration, FENO=fraction of exhaled nitric oxide. The data are presented as mean ± SEM. ^***^p<0.001,^ **^p<0.01, and ^*^p<0.05.

The untargeted lipidomics analysis included 24 asthma subjects and 20 HC subjects as previously described 21. As **Fig. 1A** indicates, 23 sphingolipids changed in asthma patients (|Fold change|≥1) compared to HCs, which was distributed in sphingosine, ceramide, sphingomyelin, etc. The correlation clustering‐heatmap indicates a clear correction between different types of sphingolipids **(Fig. 1B)**. To further investigate the sphingosine metabolism in asthma patients with different endotypes, targeted lipidomics analysis was conducted in which 16 EA patients, 15 NEA patients and 10 HCs were included. Clinical data is provided in **Table S1**. The results indicated that sphingosine and sphinganine were elevated in the plasma** (Fig. 1C)** whereas in the induced sputum it exhibited decreased in both EA and NEA patients **(Fig. 1D)**. S1P, dhS1P, and P1P, phosphorylated by sphingosine kinase (SKs), are the active terminal derivative of sphingosine metabolism 27. Plasma concentrations of S1P, dhS1P, and P1P were increased in both disease groups (P1P not shown) while induced sputum levels of these phosphorylated sphingosine metabolites were too low to detect **(Fig. 1E)**. Subsequently, we discovered that in asthma patients, the concentration of sphingosine metabolites in plasma and induced sputum were highly associated with clinical parameters of asthma **(Fig. 1F-H)** including Asthma Control Test (ACT), serum total IgE concentration, fractional exhaled nitric oxide (FENO) and index of pulmonary function FEV1/FVC (data not shown), indicating the potential impact of sphingosine metabolism on the pathogenesis of asthma.

### S1PR4 declined more in asthmatic lungs with neutrophilic inflammation

**Figure 2:**
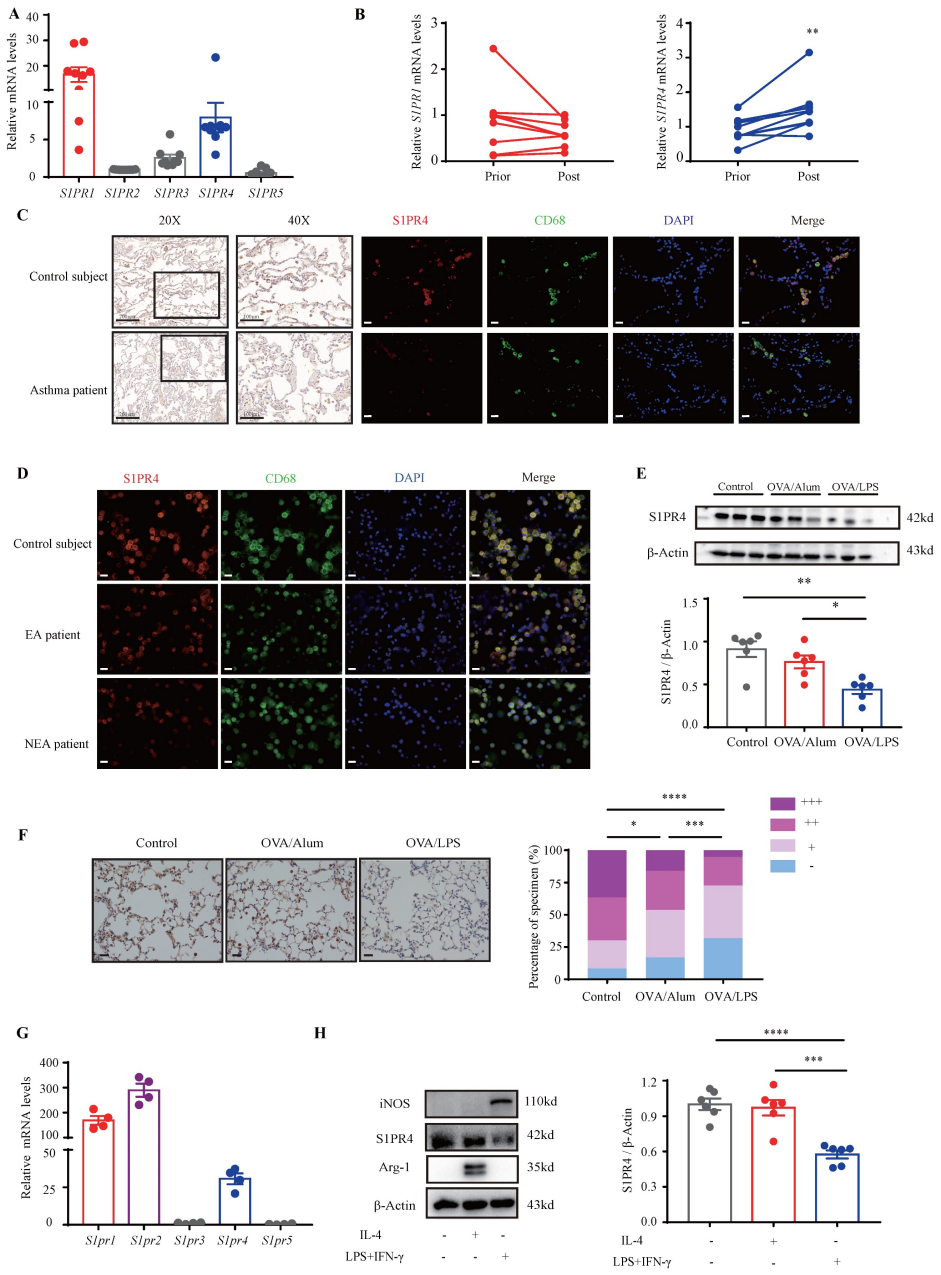
**Analysis of S1PR4 expression in asthma patients, experimental animals, and primary macrophages. A:** *S1PR*'s expression in the PBMCs of asthma patients (n=9). B: *S1PR4* expression changes in PBMC before and after standard asthma treatment. Prior=prior to standardized management and treatment of asthma, Post=after standardized management and treatment of asthma (n=6). **C:** Representative results of immunohistochemical staining of S1PR4 and co-immunostaining of S1PR4 and CD68 in the lung samples of asthma patients and control subjects. The images were captured under original magnification ×400. Scale bar, 20 μm. **D:** Representative results of co-immunostaining of S1PR4 and CD68 in the induced sputum smear from asthmatic patients with different endotypes and control subject. The images were captured under original magnification ×400. Scale bar, 20 μm. **E:** Western blot analysis of S1PR4 expression in the lungs of experimental animals (n=6). **F:** Representative images for immunohistochemical staining of S1PR4 in the lung sections of experimental animals (n=5). The images were captured under original magnification ×200. Scale bar, 50 μm. **G:** RT-PCR analyses of *S1pr*'s expression in BMDMs from WT mice (n=4). **H:** Western blot results of S1PR4 expression in BMDMs from WT mice after stimulation of IL-4 or LPS/IFN-γ (n=6). PBMC=peripheral blood mononuclear cells. BMDM=bone marrow derived macrophage. The data are presented as mean ± SEM. ^****^p<0.0001, ^***^p<0.001,^ **^p<0.01, and ^*^p<0.05.

S1P is proven to employ most of its functions in immunity through activation of its distinct GPCR 28. Moreover, it has been demonstrated that dhS1P and P1P also bind to S1PRs 14, 29. To confirm the role of S1PRs in asthma, we first investigated their expression in the PBMCs of asthmatic patients. The results showed that *S1PR1* and *S1PR4* were the main receptors expressed in asthmatic PBMCs** (Fig. 2A)**. Furthermore, the expression changes in PBMCs before and after asthmatic treatment were compared and the results indicated that *S1PR4* was the only elevated receptor after standardized management and treatment of asthma **(Fig. 2B, Fig. S1A)**. To verify these results, we examined the expression of S1PR4 in the lungs of asthma patients and HC subjects. As shown in **Fig. 2C**, S1PR4 was expressed mainly in the alveoli and the majority of macrophages. After assorting the results according to different inflammatory endotypes based on the sputum analyses, we found a surprising observation that S1PR4 declined greatly in the induced sputum smear of NEA patient **(Fig. 2D)**. We further evaluated the expression of S1PR4 in mice lungs with experimental models and demonstrated that S1PR4 exerted reduced expression in asthmatic mice which was more remarkable in the OVA/LPS treated group (**Fig. 2E-F**). Subsequently, we evaluated the receptor's expression in the primary macrophages. *S1pr1*, *S1pr2*, and *S1pr4* were mainly expressed in BMDMs **(Fig. 2G)** and *S1pr4* was obviously increased after stimulation of bioactive sphingosine metabolites **(Fig. S1B)**. Macrophages have two different forms of polarizations: either the classically activated phenotype (M1) or the alternatively activated phenotype (M2), either form is determined by the micro-environment *in vivo* or different stimulus *in vitro* 30. We found that S1PR4 was down-regulated in LPS/IFN-γ induced M1 macrophages, both in primary BMDMs **(Fig. 2H)** and lung macrophages **(Fig. S1C)**.

### S1PR4 deficiency aggravated neutrophilic inflammation in asthmatic mice

**Figure 3:**
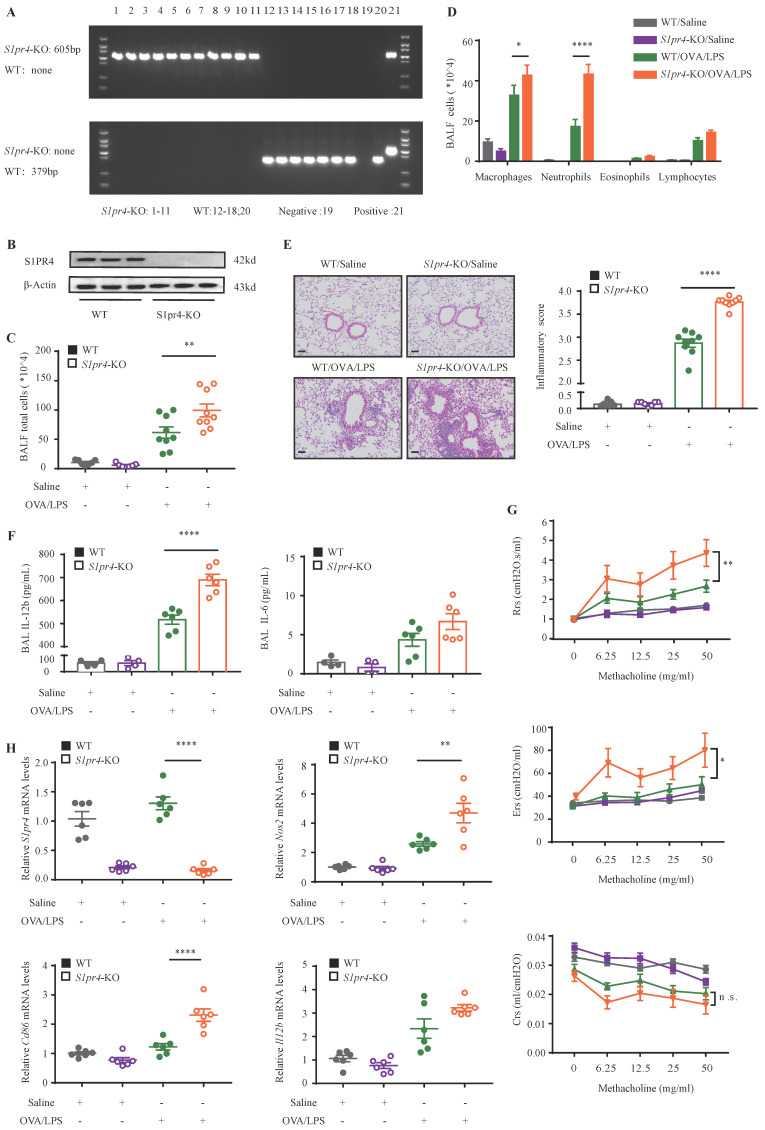
***S1pr4*-KO mice exhibited aggravated lung inflammatory response after OVA/LPS induction. A:** Genotyping results of WT and *S1pr4*-KO allele. The WT allele is none and the *S1pr4*-KO allele is 605 bp (up). The WT allele is 379 bp and the *S1pr4*-KO allele is none (below). **B:** Western blot analysis of S1PR4 expression in the lungs of *S1pr4*-KO and WT mice. **C:** Total cells, **D:** Differential counts of inflammatory cells in BALF of *S1pr4*-KO mice and WT mice after OVA/LPS induction (n=4-10). **E:** Representative images and statistical graph (n=4-10) of lung histology of *S1pr4*-KO and WT mice following OVA/LPS induction (stained with H&E). The images were captured under original magnification ×200. Scale bar, 50 μm. **F:** ELISA analysis of IL-6 and IL-12b levels measured in BALF (n=4-10). **G:** Effects of *S1pr4* deficiency on AHR, including Rrs, Ers, Crs measured 24 h following the final challenge with flexiVent (n=4-10). **H:** RT-PCR results for *S1pr4*, *Nos2*, *Cd86* and *Il12b* expression in the lungs of *S1pr4* deficiency mice following OVA/LPS induction (n=4-10). H&E= hematoxylin & eosin. Rrs=respiratory system resistance. Ers=respiratory system elastance. Crs= respiratory system compliance. The data are presented as mean ± SEM. ^****^p<0.0001, ^**^p<0.01, and ^*^ p<0.05. n.s.= no significance.

On the basis of the findings above, we aimed to dissect the effect of S1PR4 on asthma by treating *S1pr4*-KO and WT mice **(Fig. 3A-B)** with OVA/LPS induction. Compared with WT mice, *S1pr4*-KO mice showed more inflammatory cell infiltration** (Fig. 3C)**, especially macrophages and neutrophils **(Fig. 3D)**. Predictably, the severity of inflammatory response following OVA/LPS induction was significantly elevated in *S1pr4*-KO mice **(Fig. 3E)**. Next, we compared inflammatory cytokine levels in BALF measured by ELISA** (Fig. 3F)** and significantly increased IL-12b levels were observed in *S1pr4*-KO mice treated with OVA/LPS while IL-6 secretion also tended to be enhanced by *S1pr4* deficiency (the concentration of IL-1β was undetectable in most mice). Airway hyperresponsiveness (AHR) is proven to be an important feature of asthma. Therefore, we measured the respiratory system resistance (Rrs), airway tissue elasticity (Ers), and compliance (Crs) to aerosolized methacholine by applying an invasive method 24 h following the last challenge which was performed by using the flexiVent setup (SCIREQ, Canada). We demonstrated that *S1pr4* deficiency produced significant enhancement in resistance and elasticity parameters** (Fig. 3G)**.

To support the above findings, mice were infected with adenovirus expressing *S1pr4* (*S1pr4*-OE) or control vector **(Fig. S2A)**. Inversely, significantly attenuated pulmonary inflammatory response and less inflammatory cell infiltration were noted in *S1pr4*-OE mice following OVA/LPS treatment **(Fig. S2B-D)** while proinflammatory cytokines and parameters reflecting airway responsiveness showed no significant changes **(Fig. S2E-F)**. We further explored the impact of S1PR4 on eosinophilic asthma. The results showed that *S1pr4*-KO had no effect on eosinophilic airway inflammation after OVA/Alum induction **(Fig. S3A-D)**. Type-2 (T2) inflammatory response plays an important role in eosinophilic asthma. We observed that T2 cytokines had the same expression in *S1pr4*-KO mice and WT mice after OVA/Alum induction **(Fig. S3E)**.

### Deficiency of S1PR4 facilitated proinflammatory macrophage activation

**Figure 4:**
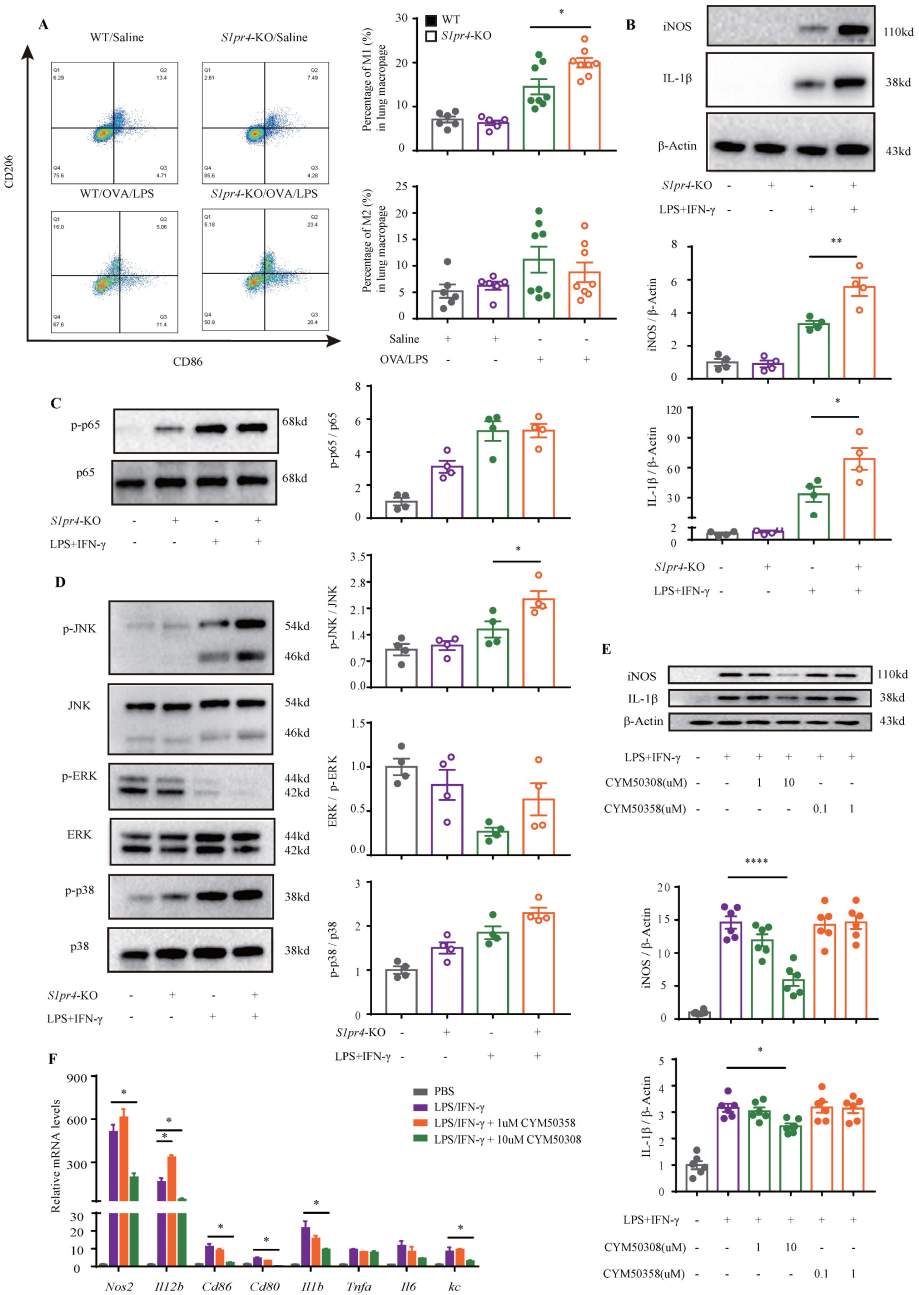
** S1PR4 restrains macrophage M1 program concerned with activated JNK pathway. A:** Flow cytometry analysis of macrophages obtained from lung tissues of *S1pr4*-KO and WT mice following OVA/LPS induction. M1=CD45^+^ CD11b^+^ F4/80^+^ CD86^+^ CD206^-^ macrophages, M2=CD45^+^ CD11b^+^ F4/80^+^ CD86^-^ CD206^+^ macrophages (n=4-10). **B:** Western blot results of the expression of iNOS and IL-1β in BMDMs generated from *S1pr4*-KO and WT mice subjected to LPS/IFN-γ stimulation (n=4). **C-D:** Western blot results of the function of S1PR4 in activating the NF-κB (C) and MAPKs pathways (D) in BMDMs generated from *S1pr4*-KO and WT mice subjected to LPS/IFN-γ stimulation (n=4). **E:** Western blot results of the effect of CYM50308 and CYM50358 on iNOS and IL-1β expression in BMDMs generated from WT mice (n=6). **F:** RT-PCR analysis of the effect of CYM50308 and CYM50358 on the target genes connected with M1 macrophage polarization in BMDMs generated from WT mice (n=6). The data are presented as mean ± SEM. ^****^p<0.0001, ^**^p<0.01, and ^*^ p<0.05.

Since primary macrophages stimulated with LPS/IFN-γ were associated with a reduced expression of S1PR4, we evaluated whether S1PR4 participated in regulating the functionalities of proinflammatory macrophages. First, we conducted RT-PCR analysis of indexes of M1 macrophage polarization. *S1pr4*-KO mice had significantly higher nitric oxide synthase 2 (*Nos2*) and *Cd86* mRNA expression after OVA/LPS induction **(Fig. 3H)**. This result was also established by flow cytometry analysis of single cell suspension of mice lung tissue. *S1pr4*-KO mice exhibited a significantly enhanced percentage of lung M1 macrophages (CD45^+^CD11b^+^F4/80^+^CD86^+^ CD206^-^) than WT mice, however, it seems unlikely that *S1pr4* deficiency affected the induction of M2 macrophages as we were not able to detect an evident difference in the number of CD45^+^CD11b^+^F4/80^+^CD206^+^ CD86^-^ macrophages **(Fig. 4A)**. RT-PCR analysis of the two M1 markers in *S1pr4*-OE mice following OVA/LPS treatment demonstrated corresponding results **(Fig. S4A)**, while the percentage of lung M1 macrophages remained unchanged **(Fig. S4B)**.

To further clarify the mechanism responsible for S1PR4 regulation of the M1 macrophage program, BMDMs and primary lung macrophages generated from *S1pr4*-KO and WT mice were applied. LPS/IFN-γ induced significantly higher levels of iNOS and IL-1β protein **(Fig. 4B)** as well as pro-inflammatory cytokines release in *S1pr4*-KO BMDMs than in WT BMDMs **(Fig. 7D)**, and a substantial increase in expression of M1 macrophages related to the pro-inflammatory gene was noted upon LPS/IFN-γ stimulation in *S1pr4*-KO primary lung macrophages **(Fig. S5)**. Given that S1PR1 and S1PR2 are closely related to NF-κB 31, 32 and the mitogen-activated protein kinases (MAPKs) pathway 33, 34, we further examined whether S1PR4 is associated with the activation of these pathways. *S1pr4*-KO induced remarkable phosphorylation of c-Jun NH2-terminal kinase (JNK) instead of other pathways (**Fig. 4C-D)**, indicating to the possible role of JNK pathway as a downstream of S1PR4. Consistently, loss of *S1pr4* also significantly promoted the expression of p-JNK in asthmatic experimental mice **(Fig. 7C)** whereas the impact was inconspicuous in *S1pr4*-OE mice **(Fig. S4C).** Subsequently, S1PR4 modulators were applied to supported the findings above. As shown in **Fig. 4E-F**, the S1PR4 agonist CYM50308 could significantly inhibit M1 macrophage polarization in both western blot and RT-PCR analyses, whereas the S1PR4 antagonist CYM50358 seemed to have an insignificant impact.

### FPR2 signaling mediate S1PR4-induced M1 macrophage programming

**Figure 5:**
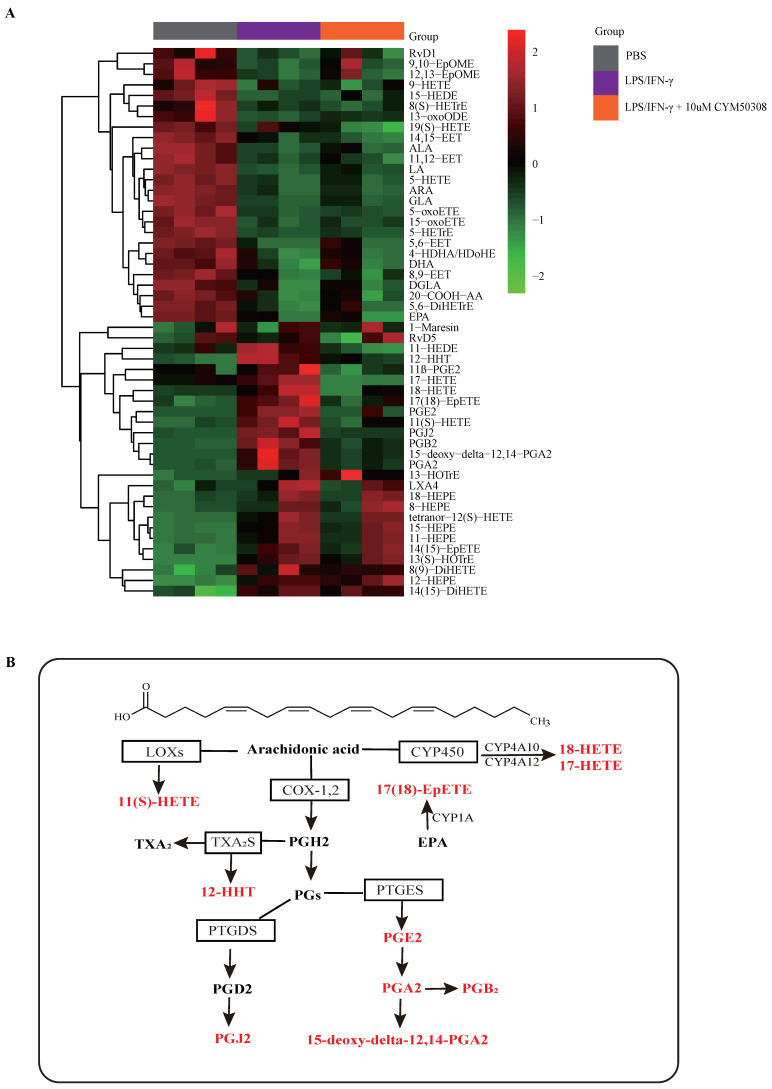
** The impact of S1PR4 on proinflammatory oxylipin's metabolic pathway. A:** Heatmap analysis of various proinflammatory oxylipins derived from AA, EPA, and DHA using targeted lipidomics analysis (n=4). The colors in the heatmap depict the fold enrichment in each sample. **B:** Metabolic pathway analysis of selected oxylipins change after CYM50308 treatment and their critical lipid metabolizing enzymes based on the KEGG database. AA=arachidonic acid, EPA=eicosapentaenoic acid, DHA= docosahexaenoic acid. KEGG=Kyoto Encyclopedia of Genes and Genomes.

Next, we aimed to clarify the mechanisms underlying M1 macrophage programming regulated by S1PR4. Based on previous studies indicating that S1PRs participated in the metabolic processes of oxylipins such as arachidonic acid (AA), which controls many physiological and pathological processes and are upstream of inflammatory activity 35-37, we conducted a targeted lipidomics analysis based on various oxylipins. The results showed that the concentration of bioactive oxylipins including AA, eicosapentaenoic acid (EPA), and docosahexaenoic acid (DHA) derived metabolites changed greatly after LPS/IFN-γ stimulation, while ten of them restored after treatment with CYM50308 (**Fig. 5A**), implying that S1PR4 has an impact on the oxylipins metabolic pathway. As exhibited in **Fig. 5B,** almost all of the selected oxylipins belonged to AA-derived metabolites and numerous enzymes were involved. Therefore, we detected the expression changes of these critical lipid metabolizing enzymes in *S1pr4*-KO BMDMs. Unfortunately, no apparent expression changes of the enzymes were observed (**Fig. S6**, prostaglandin D2 synthase was undetectable).

**Figure 6:**
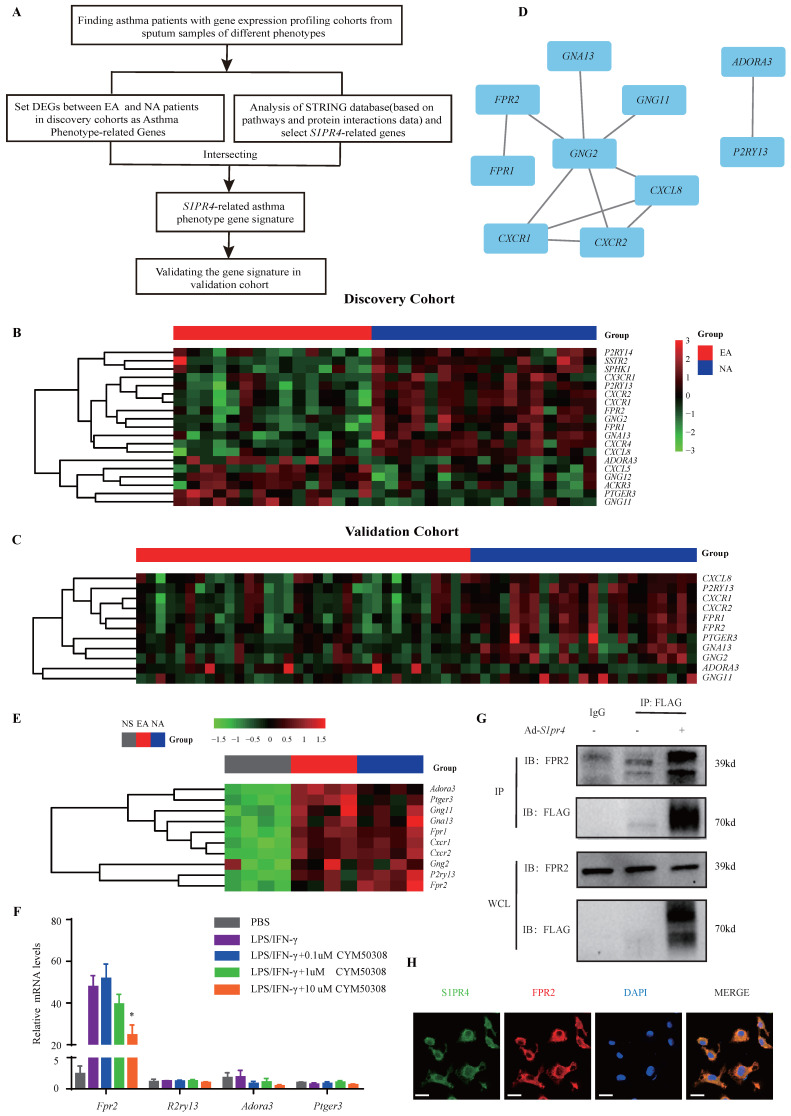
** FPR2 works as S1PR4-related asthma phenotype gene. A:** Flow diagram of comprehensive bioinformatics analysis. **B:** Heatmap analysis of discovery cohort GSE41863. The colors in the heatmap depict the fold enrichment in each sample. **C:** Conjoint analysis of GSE45111 and GSE137268 as validation cohort and gene expression in heatmap. The colors in the heatmap depict the fold enrichment in each sample. **D:** Network diagram of interrelated DEGs in the validation cohort by Cytoscape. **E:** RT-PCR analysis of DEGs in asthmatic experimental mice and presented in heatmap (n=4). The colors in the heatmap depict the fold enrichment in each sample. **F:** RT-PCR results for selected genes expression in BMDMs stimulated with different concentrations of CYM50308 (n=4). **G:** Coimmunoprecipitation and western blot results of FPR2 and FLAG in adenovirus (Ad)-treated BMDMs generated from WT mice. **H:** Laser confocal double immunofluorescence staining of FPR2 and S1PR4 in BMDMs from WT mice. The images were captured under original magnification ×1000. Scale bar, 100 μm. NS=control, EA= eosinophilic asthma, and NA= neutrophilic asthma. DEGs= differentially expressed genes. WCL=whole cell lysate. The data are presented as mean ± SEM. ^*^p<0.05.

**Figure 7:**
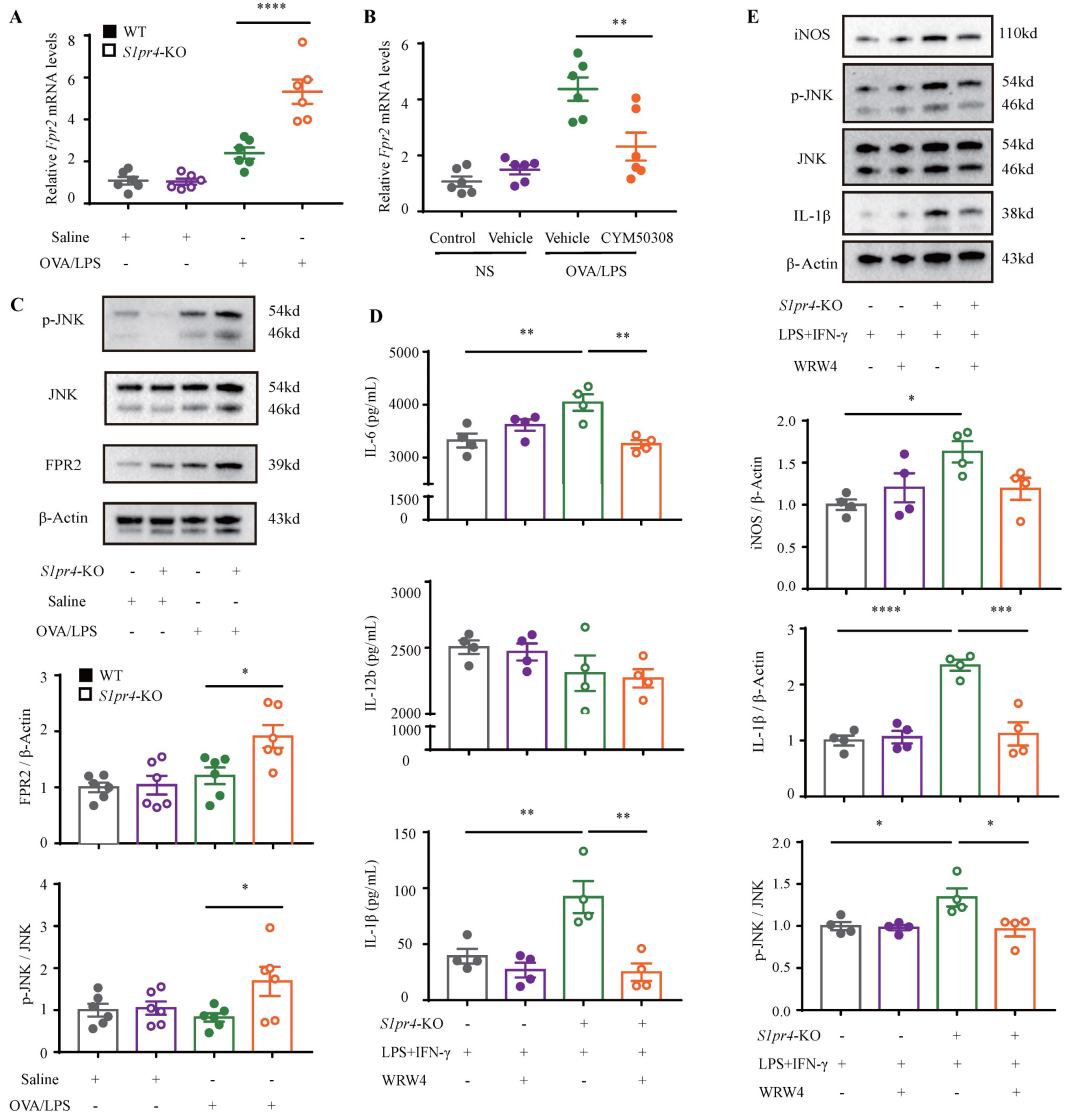
** S1PR4 interacts with FPR2 participating in macrophage M1 program. A-B:** RT-PCR results for *Fpr2* expression in the lungs of *S1pr4* deficiency (A) and CYM50308 administration (B) mice following OVA/LPS induction (n=6). **C:** Western blot analysis of the JNK pathway and FPR2 expression in the lungs of *S1pr4* deficiency mice after OVA/LPS induction(n=6). **D:** ELISA analysis of IL-6, IL-12b and IL-1β levels in *S1pr4*-KO BMDMs treated with WRW4 before LPS/IFN-γ stimulation (n=4). **E:** Western blot results of the iNOS, JNK pathway, and IL-1β expression in *S1pr4*-KO BMDMs treated with WRW4 before LPS/IFN-γ stimulation (n=4). The data are presented as mean ± SEM. ^****^p<0.0001, ^***^p<0.001,^ **^p<0.01, and ^*^p<0.05.

Subsequently, we filtered out gene arrays concerned with asthma endotypes in the GEO database followed by a comprehensive bioinformatics analysis as **Fig. 6A** shows. Nineteen genes were screened out (**Fig. 6B)** in the discovery cohort (GSE41863) and most of them were also DEGs in the validation cohort (GSE45111 and GSE137268)** (Fig. 6C).** Further analyses of the interrelated DEGs (**Fig. 6D)** were performed in asthmatic experimental mice (**Fig. 6E)** and eventually verified in BMDMs stimulated with different concentrations of CYM50308 (**Fig. 6F)**. *FPR2* was the only gene which satisfied the requirements above. We also detected its expression in *S1pr4*-KO mice (**Fig. 7A, C**) and BMDMs (**Fig. S6**) and significant up-regulated expression was observed. Co-Immunoprecipitation (**Fig. 6G**) and laser confocal double immune-fluorescence staining (**Fig. 6H**) were conducted to prove the interaction between S1PR4 and FPR2 in BMDMs. Finally, the FPR2 antagonist WRW4 was applied to reaffirm that FPR2 signaling played a part in S1PR4-induced M1 macrophage programming. Decline in parameters of macrophage polarization, phosphorylation of JNK, as well as proinflammatory cytokines release were observed in *S1pr4*-KO BMDMs treated with WRW4 following stimulating by LPS/IFN-γ (**Fig. 7D-E**). Overall, the results of this study support the hypothesis that FPR2 signaling participates in S1PR4-induced M1 macrophage programming.

### S1PR4 agonist evokes an anti-inflammatory response connected with reduced M1 macrophages

**Figure 8:**
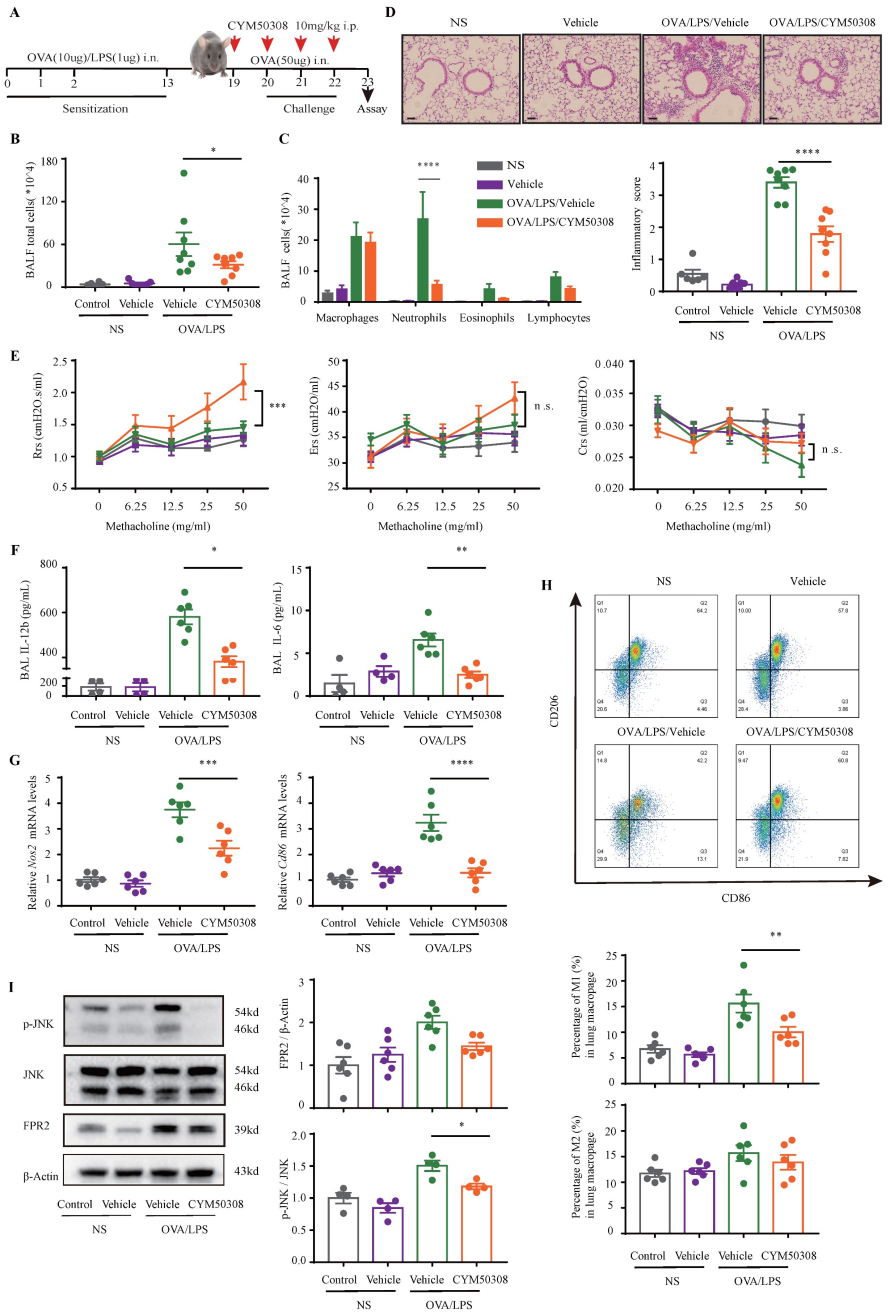
** Administration of CYM50308 shielded mice from OVA/LPS-induced neutrophilic airway inflammation. A:** The schematic diagram of CYM50308 administration. **B:** Total cells, **C:** Differential counts of inflammatory cells in BALF of CYM50308-treated and control mice after OVA/LPS induction (n=4-10). **D:** Representative images and statistical graph (n=4-10) of lung histology of CYM50308-treated and control mice following OVA/LPS induction (stained with H&E). The images were captured under original magnification ×200. Scale bar, 50 μm. **E:** AHR of CYM50308-treated and control mice reflected by Rrs, Ers and Crs (n=4-10). **F:** ELISA analysis of effects of CYM50308 treatment on the cytokine production levels of IL-12b and IL-6 in BALF (n=4-10). **G:** RT-PCR results for *Nos2* and *Cd86* expression in the lungs following OVA/LPS induction (n=4-10). **H:** Flow cytometry analysis of macrophages obtained from lung tissues of CYM50308-treated and control mice following OVA/LPS induction(n=4-10). **I:** Western blot results of JNK pathway and FPR2 expression in the lungs of CYM50308 administered mice after OVA/LPS induction(n=4-10). The data are presented as mean ± SEM. ^****^p<0.0001, ^***^p<0.001, ^**^p<0.01, and^ *^p<0.05.

Finally, we aimed to facilitate the translation of above findings to a therapeutic approach for NEA. For this purpose, experimental mice were administered with S1PR4 agonist CYM50308 or equal volume SBE-β-CD the day before challenge and each time prior to challenge starting on day 19 **(Fig. 8A)**. CYM50308 treatment significantly weakened the pulmonary inflammatory response as well as immune cell infiltration, particularly of neutrophils **(Fig. 8B-D)**. The treatment-group mice showed a decline in resistance parameters **(Fig. 8E)** while Ers and Crs remained no significant changes. Additionally, the inflammatory cytokines levels found in BALF were consistent with the assessment of inflammation levels above (the concentration of IL-1β was undetectable in most mice)** (Fig. 8F)**. Consistently, administered with CYM50308 showed significantly lower levels of *Nos2* and *Cd86* expression in the lungs **(Fig. 8G)**, combined with a significant decrease in the percentage of lung M1 macrophages **(Fig. 8H)**. Moreover, the expression of FPR2 and the phosphorylation of JNK** (Fig. 7B, Fig. 8I)** were also declined as expected. Whereas, CYM50308 seemed had marginal effect on OVA/Alum-induced eosinophilic airway inflammation (**Fig. S7A-E**).

## Discussion

Asthma is a heterogeneous pulmonary disease comprised of several different endotypes which depict various pathways of the inflammatory and immune response 38. In general, most of the current research concentrates on eosinophilic airway inflammation, with poor knowledge of principal causes and mechanisms related to non-eosinophilic phenotypes, which are less receptive to inhaled corticosteroids (ICS), the current treatment of choice for asthma 39-41.

**Figure 9:**
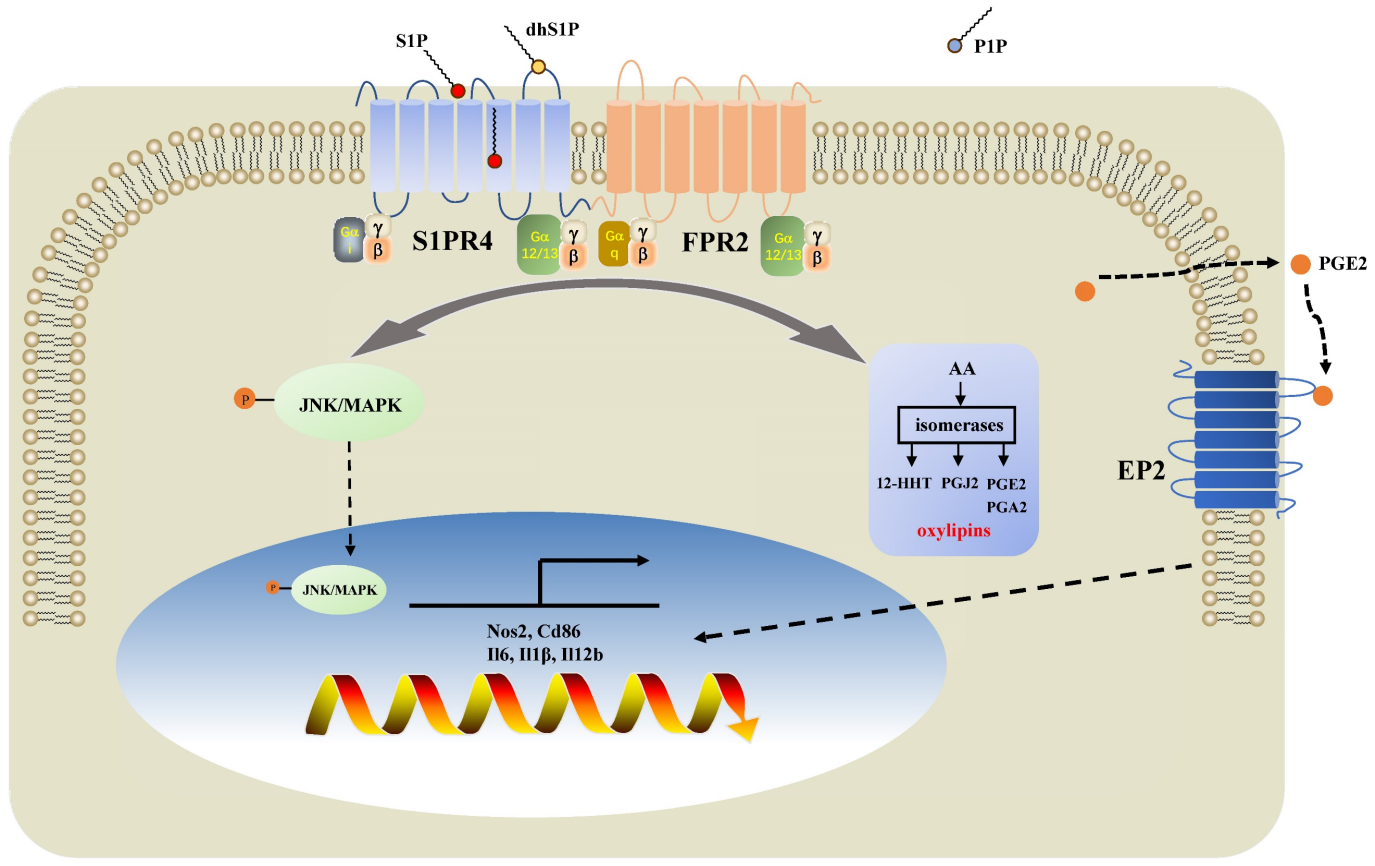
** Diagram of the mechanisms underlying S1PR4 regulation of neutrophilic asthma.** S1PR4 was strongly connected to bioactive oxylipins concurrent with bounding to FPR2 to influence the phosphorylation of JNK and then contributed to the macrophage M1 program, which in turn secreted amounts of inflammatory cytokines associated with the inflammatory response of neutrophilic asthma.

In the present study, we demonstrated that sphingolipid metabolism is abnormal in asthmatic patients, whereas sphingosine metabolites, altered largely in the untargeted lipidomics analysis, failed to distinguish between the different endotypes of asthma both in plasma and induced sputum in the targeted lipidomics analysis. We conducted further experiments with patients and mice to elucidate the impact of S1PR4, a GPCR for bioactive sphingosine metabolites, on the pathogenesis of asthma. We demonstrated that S1PR4 was mainly expressed in macrophages while it exhibited lower expression both in patients and experimental mice with neutrophilic airway inflammation. Specifically, S1PR4 was down-regulated in LPS/IFN-γ induced primary M1 macrophages while no changes were observed in M2 macrophages. Subsequently, we evaluated the function of S1PR4 in experimental mice and found that *S1pr4* deficiency aggravated asthmatic mice with neutrophilic inflammation as characterized by a reduction in M1 macrophages. Mechanistic studies showed that S1PR4 was strongly connected to bioactive oxylipins mainly generated from AA while no apparent changes in expression of the relevant enzymes were observed in *S1pr4*-KO BMDMs. Further research showed that S1PR4 bound to FPR2 to influence the phosphorylation of JNK and then contributed to the macrophage M1 program, which in turn led to the secretion of a great number of inflammatory cytokines involved in the inflammatory response of neutrophilic asthma endotype (**Fig. 9)**.

An increasing amount of evidence suggests that S1P and its receptors are relevant to the onset and development of inflammatory disorders including asthma, multiple sclerosis, inflammatory bowel disease, and rheumatoid arthritis 13, 42. It has been reported that systemic administration of S1P induced the pro-remodeling response, likely acting through S1PR2 and S1PR3 receptors 43-45. In addition, S1P may be involved in stimulating C‐fiber‐mediated airway sensations and reflexes via S1PR3 receptors 46. Furthermore, S1PR2 is involved not only in mast cell degranulation but also in regulating CCL3 production in airway epithelial cells, while its antagonist JTE-013 reduces the inflammatory cell infiltration possibly by regulating autophagy through RAC1 47-49. S1PR1 has great effect on lymphatic recirculation 50 related to lymphopenia 51, though it has been linked to regulating Th2 cell migration 52, 53. In our study, *S1PR1* and *S1PR4* were the main receptors expressed in asthmatic PBMCs, while *S1PR4* was the only elevated receptor after standardized management and treatment of asthma, indicating to a predictable effect on the pathophysiology of asthma, especially in the immune response.

Macrophages, the most commonly found immune cells in the lung, are vital elements of innate and adaptive immunity and have a significant impact on tissue homeostasis 54. Both of M1/M2 macrophages have been associated with the pathogenesis of asthma 55-57. In our study, *S1pr4*-KO mice had significantly enhanced M1 program with no impact on the induction of M2 macrophages after OVA/LPS induction. On the contrary, mice administered with CYM50308 had much lower levels of *Nos2* and *Cd86* expression combined with a significant decrease in the percentage of lung M1 macrophages. Given that M1 macrophages assemble a large number of pro-inflammatory Th1 cytokines (e.g. IL-6, IL-12, IL-1β, etc.) and chemokines (e.g. CCL2, CCL5, etc.), indicative of a leading function in the recruitment and activation of Th1 cells as well as clearance of intracellular pathogens, it has been referred to as the potential major effector in non-allergic asthma and associated with the pathophysiology of severe asthma, especially in those with a poor reaction to systemic corticosteroids 58, 59. We further investigated whether S1PR4 had an impact on the polarization of M1 macrophages and its potential mechanism. We demonstrated that BMDMs and primary lung macrophages derived from *S1pr4*-KO mice were shown to promote the functionalities of proinflammatory macrophages upon LPS/IFN-γ stimulation, which were further confirmed by S1PR4 agonist CYM50308. Next, we investigated the essential question of how S1PR4 affected the M1 program in macrophages. Previously published studies have indicated that S1P inhibits the 5-lipoxygenase product formation, while the effect was simulated by S1PR4-specific agonists and confined to cells expressing S1PR4 35. Contrastingly, other studies have suggested that S1P induces cyclooxygenase-2 activity and the consequential assembly of PGE2, which may occur via S1PR2 60-62. We therefore studied the impact of S1PR4 on oxylipin's metabolism and related signaling. Indeed, bioactive oxylipins changed greatly after M1 polarization, while some of AA-derived metabolites restored after treatment with CYM50308. Unfortunately, no apparent changes in the expression of enzymes including PG synthetase, cyclooxygenase, and lipoxygenase were observed in *S1pr4*-KO BMDMs. Lastly, we selected FPR2, which serves as the key connection between S1PR4 and the M1 program by means of bioinformatics analysis. FPR2 is a member of the GPCR family expressed by many cells including most if not all immune cells. It attaches to several types of ligands consisting of chemotactic formyl peptides, bioactive lipid metabolites of AA or DHA (lipoxin A4 and resolvin D1, respectively), as well as urokinase-type plasminogen activator receptor 63-65. A decline in M1 polarization, phosphorylation of JNK, as well as proinflammatory cytokines release were observed in *S1pr4*-KO BMDMs treated with the FPR2 antagonist WRW4, which further elucidates the role of FPR2 signaling in S1PR4-induced M1 macrophage programming.

FTY720 (known as Fingolimod or Gilenya) functions as a high-affinity agonist of four of the five known S1PRs (except for S1PR2) and employs strong anti-inflammatory effects, received approval in 2010 to be used as immunomodulatory drug for multiple sclerosis 66, 67. Research shows that FTY720 inhibited the OVA or house dust mite-induced AHR in mice along with a decline in the eosinophil and lymphocyte count in BALF, which is possibly related to altered dendritic cell function 12, 68. However, as a remarkably high affinity ligand for S1PR1, FTY720 controls T cell movements at numerous phases of T cell development and responses and it was reported that extended systemic treatment of multiple sclerosis patients exacerbated their asthma 69. Although expressed abundantly in T cells, S1PR4 has relatively insignificant roles in T cell survival 70, indicating to the application prospect of S1PR4 modulator in inflammatory diseases. However, demonstration of S1PR4 function in eosinophilic asthma models has been controversial and may vary with model or genetic background of the mice 71, 72. In particular, Jeon et al used S1PR4 antagonist CYM50358 on a BALB/c background and demonstrated an anti-inflammatory response in OVA-induced mouse model of asthma 71. Our experiments were on a C57BL/6 background and results showed that *S1pr4*-KO as well as S1PR4 agonist CYM50308 had limited role on OVA/Alum-induced eosinophilic airway inflammation. It is possible that differences are observed due to inflammatory environment skew in distinct populations or from differences in protocols. What is more, in our study, administered CYM50308 *in vivo* significantly weakened the airway inflammatory response as well as AHR, while no significant decline of lymphocytes was observed. As a result, administration of CYM50308 protected mice from OVA/LPS induced neutrophilic inflammation in asthma. Despite the promising results of our study, more studies are needed for dosage and drug safety evaluation.

A few limitations exist in our study that should be addressed. Firstly, we employed a relatively small asthma cohort and more asthma patients with different endotypes are needed to verify the disturbance of sphingosine metabolism as well as the expression of S1PR4. Secondly, although we demonstrated that S1PR4 interacted with FPR2 and exerted an effect on the M1 program, the specific underlying mechanisms should be explored further. Thirdly, we illustrated that S1PR4 has an impact on inflammation-associated oxylipin's metabolism, but in-depth studies are needed between S1PR4 and related metabolic enzymes. Lastly, further research is necessary to completely elucidate the effects of S1PR4 on the function of other immune cells in the pathogenesis of asthma.

In summary, we have demonstrated that a disturbance in sphingosine metabolism existed in asthma patients independent of different endotypes while the crucial sphingosine receptor S1PR4 was altered in patients and experimental mice, especially those with neutrophilic airway inflammation. Therefore, mice deficient in *S1pr4* had aggravated OVA/LPS-induced lung inflammatory response and AHR. Mechanistic studies revealed that S1PR4 interacted with FPR2 to influence the JNK pathway and following macrophage M1 program. Oxylipin's metabolism including PG may be involved in this process. Moreover, administration of the S1PR4 agonist CYM50308 substantially protected mice from OVA/LPS-induced lung inflammation. In brief, the findings of this study substantiate the hypothesis that targeting S1PR4 may be a promising therapeutic approach for non-eosinophilic phenotypes (refractory or ICS-insensitive) of asthma in clinical settings.

## Supplementary Material

Supplementary figures and tables.

## Abbreviations

S1PR4: sphingosine-1-phosphate receptor 4
S1P: sphingosine-1-phosphate
dhS1P: dihydro-S1P
P1P: phytosphingosine-1-phosphate
GPCR: G protein-coupled receptor
FPR2: formyl peptide receptor 2
JNK/MAPK: c-Jun NH2-terminal kinase / mitogen-activated protein kinases
HC: healthy control
EA: eosinophilic asthma
NEA: non-eosinophilic asthma
NA: neutrophilic asthma
PBMC: peripheral blood mononuclear cells
OVA: ovalbumin
LPS: lipopolysaccharide
Alum: Aluminum hydroxide
FENO: fractional exhaled nitric oxide
IFN-γ: Interferon-gamma
IL-: Interleukin-
BMDM: bone marrow derived macrophage
GEO: Gene Expression Omnibus
DEG: differentially expressed gene
KEGG: Kyoto Encyclopedia of Genes and Genomes
SEM: standard error of the mean
AHR: Airway hyperresponsiveness
Rrs: respiratory system resistance
Ers: airway tissue elasticity
Crs: airway tissue compliance
BALF: bronchoalveolar lavage fluid
*S1pr4*-KO: *S1pr4* knockout
WT: wild type
*S1pr4*-OE: adenovirus expressing *S1pr4*
Nos2: nitric oxide synthase 2
AA: arachidonic acid
EPA: eicosapentaenoic acid
DHA: docosahexaenoic acid
PG: prostaglandin
ICS: inhaled corticosteroids
