# Supplementary material for: Sphingosine-1-Phosphate Receptor 4 Attenuates Neutrophilic Airway Inflammation in Experimental Asthma via Repressing Proinflammatory Macrophage Activation
Source: Int J Biol Sci. 2023 Mar 5;19(5):1597–615. doi: 10.7150/ijbs.80256 (PMC10086758; doi:10.7150/ijbs.80256)
Supplement: Supplementary file 1 — Supplementary figures and tables. [file ijbsv19p1597s1.pdf]

Sphingosine-1-Phosphate Receptor 4 Attenuates Neutrophilic Airway Inflammation In Experimental Asthma Via Repressing Proinflammatory Macrophage Activation

Shanshan Wang [1], Zhen Tian [1], Yanjiao Lu [1], Zhenli Huang [1], Yan Fan [1], Boyu Li [1], Hongyan, Zheng [1], Xiaojie Wu [2], Meijia Wang [1*], Jianping Zhao [1*], Jungang Xie [1*]

[1] Department of Respiratory and Critical Care Medicine, National Clinical Research Center of Respiratory Disease, Key Laboratory of Pulmonary Diseases of Health Ministry, Tongji Hospital, Tongji Medical College, Huazhong University of Science and Technology, Wuhan, Hubei, 430030, China

[2] Department of Respiratory and Critical Care Medicine, Wuhan NO.1 Hospital, Wuhan Hospital of traditional Chinese and Western Medicine, Wuhan 430022, China

[*] **Corresponding authors**： Meijia Wang (meijia.wang@hotmail.com), Jianping Zhao (Zhaojp88@126.com) and Jungang Xie (xiejjgg@hotmail.com)

Keywords: sphingosine metabolism, S1PR4, macrophage, FPR2, neutrophilic asthma

Table S1： EA, NEA Patients and HC Subjects: Clinical Characteristics

Characteristics	EA Patients	NEA Patients	HC Subjects	P-value
number	16	15	10	
Sex (F/M)	10/6	10/5	5/5	0.209
Age (y)	46.06 ± 3.01	49.73 ± 2.70	43.60 ± 4.67	0.454
BMI (kg/m^2)	23.82 ± 0.88	25.16 ± 0.91	23.10 ± 1.02	0.324
ACT	15.06 ± 0.70	15.43± 1.07	NA	0.167
ACQ	2.25 ± 0.20	1.87± 0.24	NA	0.501
Lung function				
FEV_1 (L)	2.38 ± 0.26	2.19 ± 0.13	2.80 ± 0.15	0.148
FEV1% predicted	79.99 ± 5.46	86.43 ± 4.97	93.95 ± 1.67	0.175
FVC (L)	3.61 ± 0.25	3.34 ± 0.20	3.43 ± 0.14	0.658
FVC % predicted	105.03 ± 3.05	109.22 ± 5.01	96.46 ± 2.39	0.110
FEV1 / FVC (%)	63.73 ± 3.64	66.22 ± 2.58	84.24 ± 3.06	< 0.0001*
FENO	99.94 ± 19.56	44.50 ± 13.52	NA	0.184
IgE (IU/mL)	230.76 ± 47.43	106.10 ± 29.59	NA	0.03*
Blood eosinophils (%)	7.08 ± 0.94	2.86 ± 0.82	NA	0.261
Sputum eosinophils (%)	31.36 ± 5.79	1.31 ± 0.20	NA	< 0.0001*
Blood neutrophils (%)	52.76 ± 2.33	60.78 ± 2.53	NA	0.446
Sputum neutrophils (%)	41.51 ± 5.51	58.28 ± 5.31	NA	0.65

Values are presented as mean ± SEM; BMI: body mass index; ACT: Asthma Control Test; ACQ: Asthma Control Questionnaire; FENO: fractional exhaled nitric oxide; NA: not applicable

*P <0.05

Table S2: **The primers used for each target gene**

Target Gene	Primer-F	Primer-R
S1PR1	ACCCCATCATTTACACTCTGACC	GGTTGTCCCCTTCGTCTTTCT
S1PR2	TCGTGCTAGGCGTCTTTATCG	AGTGGGCTTTGTAGAGGATCG
S1PR3	AACCCGGTCATCTACACGCTGG	GCAGGTCTTCCTTGACCTTCG
S1PR4	AACCCCATCATCTACTCCTTCC	AGCCGCGAAAGCTGTCCCTTG
S1PR5	AACCCCATCATCTACACGCTCA	AGCCGCTGAAGCTCCCATCAA
S1pr1	AAATGCCCCAACGGAGACT	CTGATTTGCTGCGGCTAAATTC
S1pr2	GCCATCGTGGTGGAGAATCTT	AGGTACATTGCTGAGTGGAACTTG
S1pr3	GAACTTTCCCGACTGCTCTACCA	TGGCGGTGAAGATACTGATGAG
S1pr4	CTTTGTGGTGTGCTGGGGTC	GCGGAAGGAGTAGATGAGAGGA
S1pr5	CATGGCTAACTCGCTGCTGAA	AGCTGTTGGAGGAGTCTTGGTT
Tnfα	CTGAACTTCGGGGTGATCGG	GGCTTGTCACTCGAATTTTGAGA
Il6	CTGCAAGAGACTTCCATCCAG	AGTGGTATAGACAGGTCTGTTGG
Nos2	GTTCTCAGCCCAACAATACAAGA	GTGGACGGGTCGATGTCAC
Il12b	GTCCTCAGAAGCTAACCATCTCC	CCAGAGCCTATGACTCCATGTC
kc	TCGAGACCATTTACTGCAACAG	CATTGCCGGTGGAAATTCCTT
Il1b	GCAACTGTTCCTGAACTCAACT	ATCTTTTGGGGTCCGTCAACT
Fpr2	CCGTCCTTTACGAGTCCTTACA	CAGGAGGTGAAGTAGAACTGGT
P2ry13	ATGCTCGGGACAATCAACACC	CCACAGTATAGAGAACCGGGA
Ptger3	CCGGAGCACTCTGCTGAAG	CCCCACTAAGTCGGTGAGC
Fpr1	TCGTTTGACCACAGTCCCTAA	CTGAACCCAATGATGAACCTGAT
Adora3	ACGGACTGGCTGAACATCAC	AGACAATGAAATAGACGGTGGTG
Gng11	CCTGCCCTTCACATCGAGG	TTGTCTCTGCAACTTGACTTCTT

Gna13	GTCCAAGGAGATCGACAAATGC	CCAGCACCCTCATACCTTTGA
Gng2	ACCGCCAGCATAGCACAAG	AGTAGGCCATCAAGTCAGCAG
Cxcr1	TCTGGACTAATCCTGAGGGTG	GCCTGTTGGTTATTGGAACTCTC
Cxcr2	ATGCCCTCTATTCTGCCAGA	GTGCTCCGGTTGTATAAGATGAC
Alox5	ACTACATCTACCTCAGCCTCATT	GGTGACATCGTAGGAGTCCAC
Alox15	GGCTCCAACAACGAGGTCTAC	AGGTATTCTGACACATCCACCTT
Ptgds	GAAGGCGGCCTCAATCTCAC	CGTACTCGTCATAGTTGGCCTC
Ptges	GGATGCGCTGAAACGTGGA	CAGGAATGAGTACACGAAGCC
Ptgs1	ATGAGTCGAAGGAGTCTCTCG	GCACGGATAGTAACAACAGGGA
Ptgs2	TTCAACACACTCTATCACTGGC	AGAAGCGTTTGCGGTACTCAT
Cyp4a12b	GGGGAGATCAGACCCAAAAGC	ATTCGTCGGTGCTGAAACCAT
Cyp1a1	CAATGAGTTTGGGGAGGTTACTG	CCCTTCTCAAATGTCCTGTAGTG
Cyp1a2	TCGGTGGCTAACGTCATTGG	GCTGTTATTCACGATGTTCAGCA
Tbxas1	TACCATAGTGACTGTGACTCTGC	GGTGCCTGATGCCCAACTT

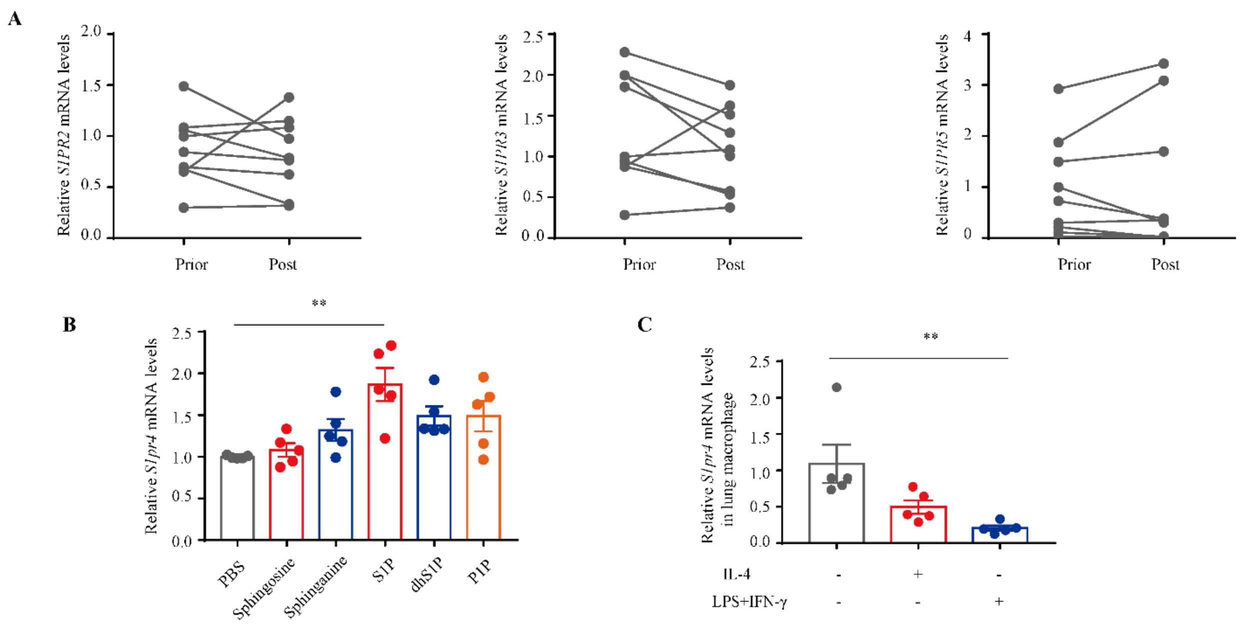

Fig. S1: S1PRs expression changes in PBMC of asthma patients and S1PR4 expression in primary lung macrophages. A: *S1PR2*, *S1PR3* and *S1PR5* expression changes in PBMC of asthma patients before and after standard asthma treatment (n=6). Prior=prior to standardized management and treatment of asthma, Post=after standardized management and treatment of asthma. **B:** RT-PCR analyses of *S1pr4* expression after stimulation of sphingosine metabolites in BMDMs (n=5). **C:** RT-PCR results of *S1pr4* expression in primary lung macrophages of WT mice after stimulation of IL-4 or LPS/IFN-γ (n=5). PBMC=peripheral blood mononuclear cells. BMDMs=bone marrow derived macrophages. S1P=sphingosine-1-phosphate, dhS1P= dihydro-S1P and P1P= phytosphingosine-1-phosphate. The data are presented as mean ± SEM. **p<0.01.

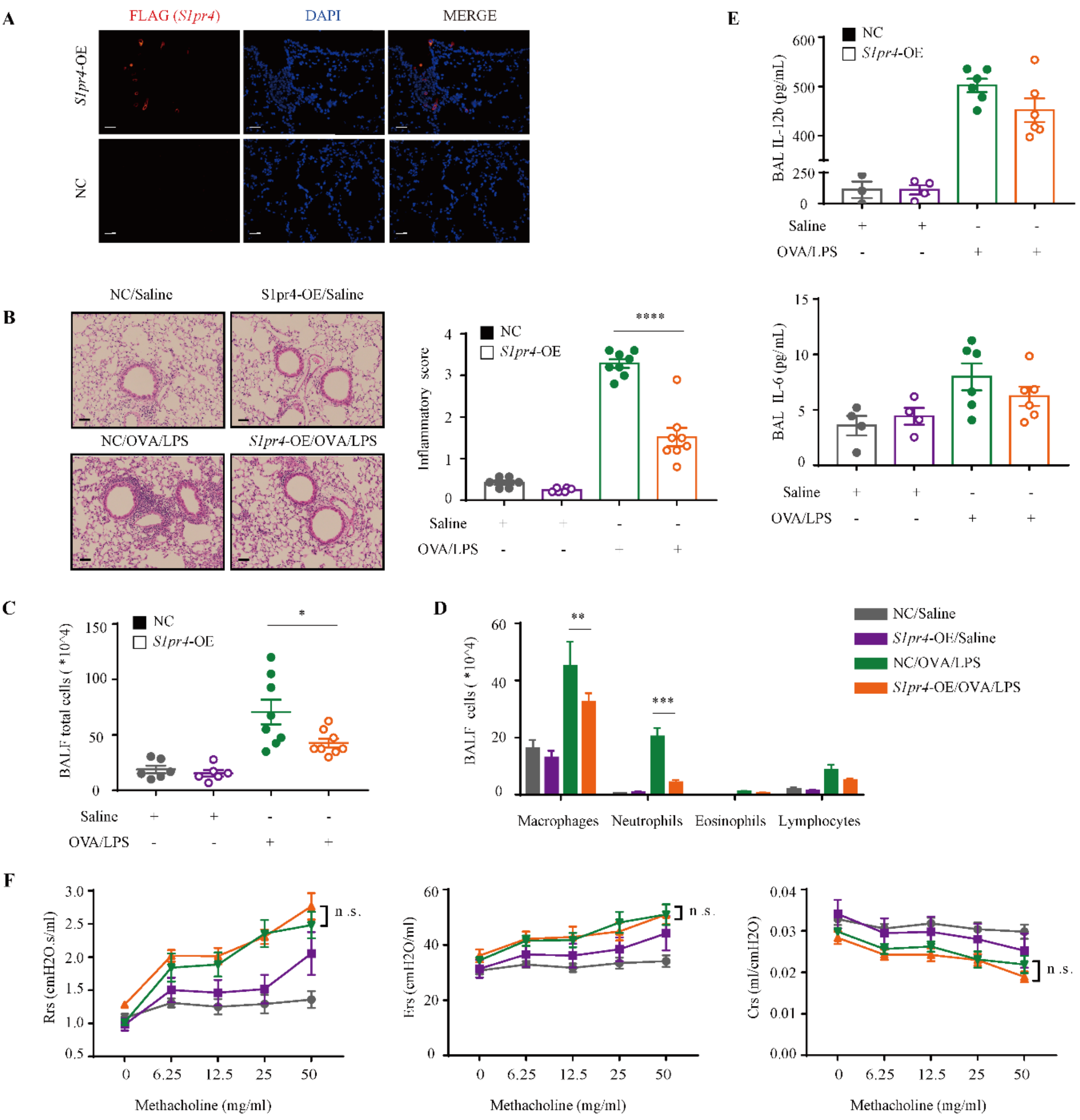

Fig. S2: ***S1pr4*-OE attenuates neutrophilic airway inflammation after OVA/LPS induction. A:** Representative results of immunostaining the FLAG in lung histology of *S1pr4*-OE and WT mice. The images were captured under original magnification×400. Scale bar, 20 μm. **B:** Representative images and statistical graph (n=4-10) of lung histology of *S1pr4*-OE and WT mice following OVA/LPS induction (stained with H&E). The images were captured under original magnification×200. Scale bar, 50 μm. **C:** Total cells, **D:** Differential counts of inflammatory cells in BALF of *S1pr4*-OE mice and control subjects after OVA/LPS induction (n=4-10). **E:** Effects of *S1pr4*-OE on cytokine production by ELISA analysis of IL-12b and IL-6 levels measured in BALF (n=4-10). **F:** AHR of *S1pr4*-OE and control mice (n=4-10). The data are presented as mean ± SEM. ****p<0.0001, ***p<0.001, **p<0.01, * p<0.05, and n.s.=no significance.

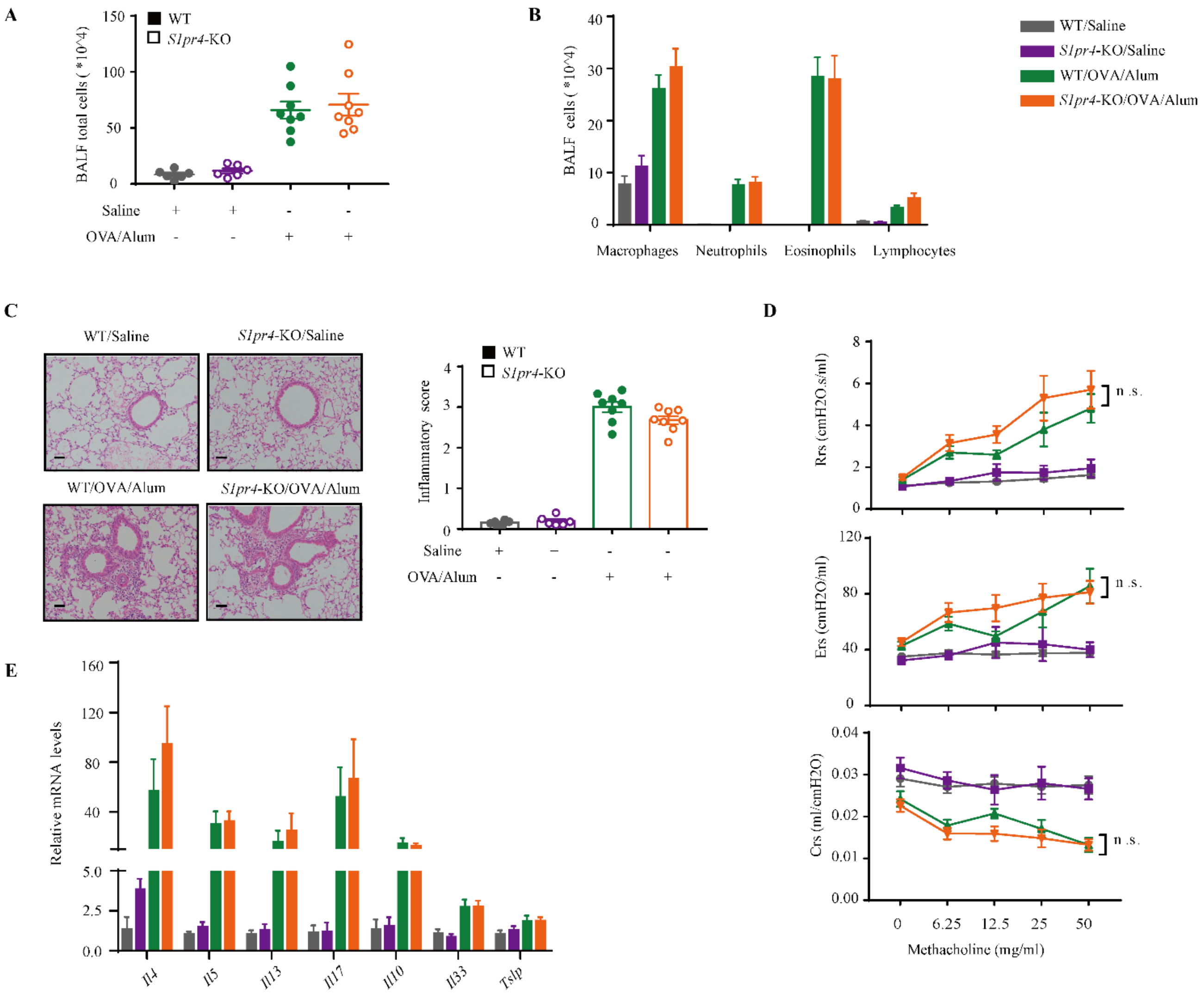

Fig. S3: *S1pr4*-KO had no effect on eosinophilic airway inflammation after OVA/Alum induction. A: Total cells, **B:** Differential counts of inflammatory cells in BALF of *S1pr4*-KO mice and control subjects after OVA/Alum induction (n=4-10). **C:** Representative images and statistical graph (n=4-10) of lung histology of *S1pr4*-KO and WT mice following OVA/Alum induction (stained with H&E). The images were captured under original magnification×200. Scale bar, 50 μm. **D:** AHR of *S1pr4*-KO and control mice (n=4-10). **E:** Effects of *S1pr4*-KO on T2 cytokine production by RT-PCR analysis measured in lung tissue after OVA/Alum induction (n=3-10). The data are presented as mean ± SEM. $^{****}p<0.0001$, $^{***}p<0.001$, $^{**}p<0.01$, * $p<0.05$, and n.s.=no significance.

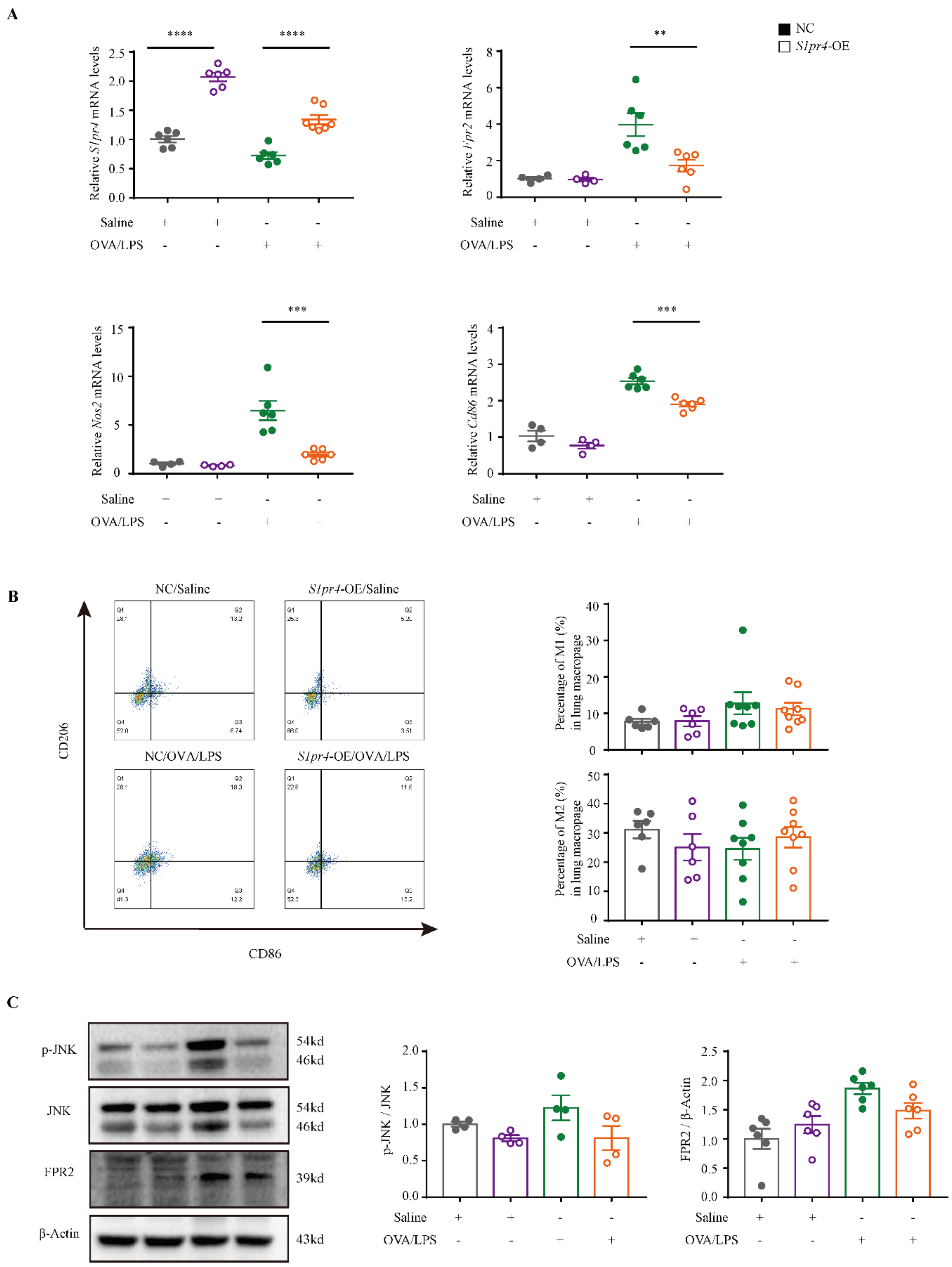

Fig. S4: *S1pr4*-OE had little effect on M1 program. A: RT-PCR results for *S1pr4*, *Fpr2*, *Nos2*, and *Cd86* expression in the lungs of *S1pr4*-OE and WT mice after OVA/LPS induction (n=4-10). **B:** Flow cytometry analysis of macrophages obtained from lung tissues of *S1pr4*-OE and WT mice after OVA/LPS induction (n=4-10). **C:** Western blot of JNK pathway and FPR2 expression in the lungs of *S1pr4*-OE and WT mice after OVA/LPS induction (n=4-10). The data are presented as mean ± SEM. ****p<0.0001, ***p<0.001 and **p<0.01.

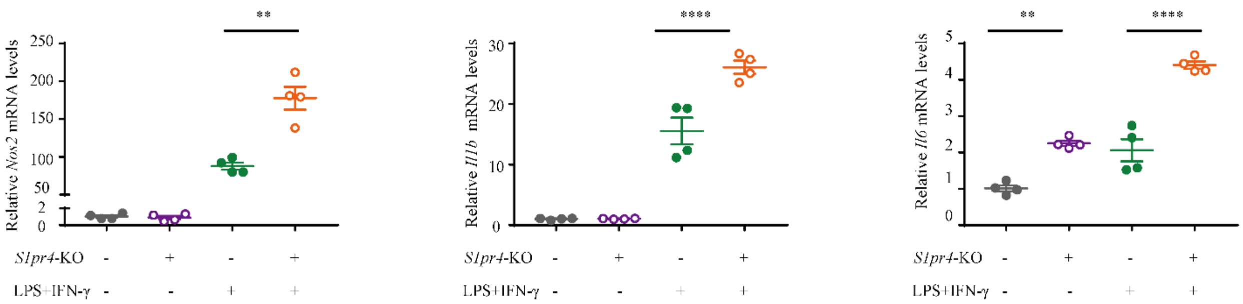

Fig. S5: RT-PCR analysis of *S1pr4* deficiency on primary lung macrophages obtained from *S1pr4*-KO and WT mice after LPS/IFN-γ stimulation (n=4). The data are presented as mean ± SEM. ****p<0.0001 and **p<0.01.

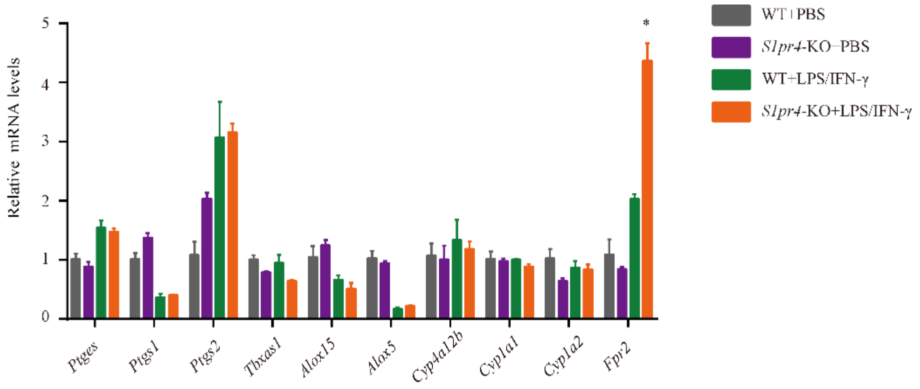

Fig. S6: The effect of *S1pr4* deficiency on lipid metabolizing enzymes. Loss of *S1pr4* failed to change the expression of lipid metabolizing enzymes connected with selected bioactive oxylipins in BMDMs generated from *S1pr4*-KO and WT mice subjected to LPS/IFN-γ stimulation (prostaglandin D2 synthase was undetectable). The data are presented as mean ± SEM. * p<0.05.

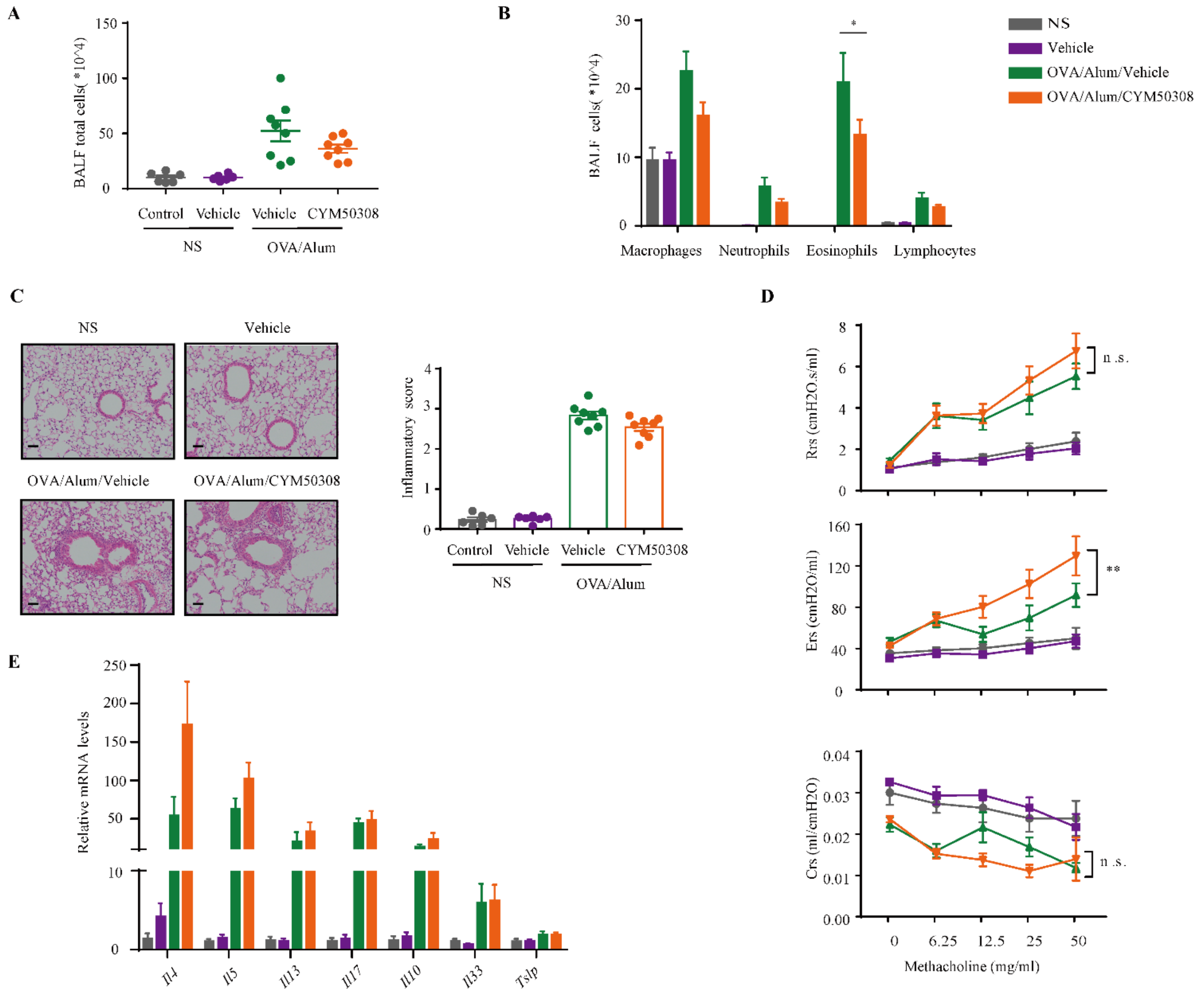

Fig. S7: Administration of CYM50308 had marginal effect on OVA/Alum-induced mice. A: Total cells, **B:** Differential counts of inflammatory cells in BALF of CYM50308-treated and control mice after OVA/Alum induction (n=4-10). **C:** Representative images and statistical graph (n=4-10) of lung histology of CYM50308-treated and control mice following OVA/Alum induction (stained with H&E). The images were captured under original magnification ×200. Scale bar, 50 μm. **D:** AHR of CYM50308-treated and control mice (n=4-10). **E:** Effects of CYM50308 on T2 cytokine production by RT-PCR analysis measured in lung tissue after OVA/Alum induction (n=3-10). The data are presented as mean ± SEM. $^{****}p<0.0001$, $^{***}p<0.001$, $^{**}p<0.01$, * $p<0.05$, and n.s.=no significance.
